# Combined inhibition of histone methyltransferases EZH2 and DOT1L is an effective therapy for neuroblastoma

**DOI:** 10.1002/cam4.70082

**Published:** 2024-11-05

**Authors:** Janith A. Seneviratne, Daenikka Ravindrarajah, Daniel R. Carter, Vicki Zhai, Amit Lalwani, Sukriti Krishan, Anushree Balachandran, Ernest Ng, Ruby Pandher, Matthew Wong, Tracy L. Nero, Shudong Wang, Murray D. Norris, Michelle Haber, Tao Liu, Michael W. Parker, Belamy B. Cheung, Glenn M. Marshall

**Affiliations:** ^1^ Children's Cancer Institute Australia for Medical Research, Lowy Cancer Research Centre UNSW Sydney Kensington New South Wales Australia; ^2^ School of Clinical Medicine, Faculty of Medicine and Health UNSW Sydney Kensington New South Wales Australia; ^3^ School of Biomedical Engineering University of Technology Sydney Sydney New South Wales Australia; ^4^ Department of Biochemistry and Pharmacology, Bio21 Molecular Science and Biotechnology Institute The University of Melbourne Parkville Victoria Australia; ^5^ Centre for Drug Discovery and Development, Clinical and Health Sciences University of South Australia Adelaide South Australia Australia; ^6^ Centre for Childhood Cancer Research UNSW Sydney Randwick New South Wales Australia; ^7^ ACRF Rational Drug Discovery Centre St. Vincent's Institute of Medical Research Fitzroy Victoria Australia; ^8^ Kids Cancer Centre Sydney Children's Hospital Randwick New South Wales Australia

**Keywords:** biomarkers, cancer biology, chromatin modifications and dynamics, epigenetics, molecular biology, neuroblastoma

## Abstract

**Background:**

The child cancer, neuroblastoma (NB), is characterised by a low incidence of mutations and strong oncogenic embryonal driver signals. Many new targeted epigenetic modifier drugs have failed in human trials as monotherapy.

**Methods:**

We performed a high‐throughput, combination chromatin‐modifier drug screen against NB cells. We screened 13 drug candidates in 78 unique combinations.

**Results:**

We found that the combination of two histone methyltransferase (HMT) inhibitors: GSK343, targeting EZH2, and SGC0946, targeting DOT1L, demonstrated the strongest synergy across 8 NB cell lines, with low normal fibroblast toxicity. High mRNA expression of both EZH2 and DOT1L in NB tumour samples correlated with the poorest patient survival. Combination HMT inhibitor treatment caused activation of ATF4‐mediated endoplasmic reticulum (ER) stress responses. In addition, glutathione and several amino acids were depleted by HMT inhibitor combination on mass spectrometry analysis. The combination of SGC0946 and GSK343 reduced tumour growth in comparison to single agents.

**Conclusion:**

Our results support further investigation of HMT inhibitor combinations as a therapeutic approach in NB.

## 1 INTRODUCTION

Drug resistance to induction cytotoxic chemotherapy agents represents an obstacle to improving neuroblastoma (NB) patient outcomes.^1^ Targeted combination therapies represent a significant benefit for patients, as they minimise the chance of acquiring resistance.^2^ Studies have shown that NB tumours have a unique oncogenic dependency on epigenetic processes, and that targeting these processes with small molecule inhibitors results in highly efficacious anticancer activity in preclinical animal models.^3^, ^4^ Epigenetic targets include histone readers, histone deacetylases (HDAC), histone methyltransferases (HMT), histone demethylases (HDM), DNA methyltransferases (DNMT) and chromatin remodelling complexes. Histone readers facilitate the reading of several histone tail modifications which controls the rate of oncogenic gene transcription. Histone readers have been effectively targeted with small molecule inhibitors in NB by disrupting super‐enhancer driven transcription.^5^ HDACs remove acetyl groups from lysine residues of histone tails, leading to chromatin compaction and altered gene transcription.^6^ HDAC inhibitors elicit anticancer efficacy against NB cells by altering gene transcription and inducing apoptotic responses.^7^, ^8^ HMTs facilitate the methylation of lysine or arginine residues on histone tails which impacts chromatin structures and modulates oncogene transcription.^9^ The inhibition of specific HMTs in NB have been demonstrated to reduce NB tumour growth.^10^, ^11^ In contrast to HMTs, HDMs remove methyl groups from histone tails, also effecting downstream gene transcription via chromatin compaction or expansion.^12^ Targeting HDMs with small molecule inhibitors in NB had also been proven to be an effective preclinical therapeutic strategy.^13^ Apart from histone modifiers, DNMTs specifically catalyse the addition of methyl groups to cysteine residues in DNA, silencing the expression of genes through promoter‐specific hypermethylation.^14^ Targeting DNMT's in NB induces differentiation by restoring tumour suppressor gene expression.^15^ Finally, chromatin remodelling complexes interact with nucleosomes and histone dimers to facilitate nucleosome disassembly upon gene transcription. Small molecule targeting of these complexes has proven to be very effective against NB tumour models.^16^ Given the effectiveness of many epigenetic‐targeted inhibitors in NB, there is high potential for synergies with other targeted agents.

There are several deregulated kinases that exacerbate pro‐tumoural signalling pathways in NB. *ALK* is the most prominent receptor tyrosine kinase to be dysregulated in NB, due to somatic or germline mutations that result in hyperphosphorylation and constitutive kinase activity of ALK proteins that drive tumorigenicity.^17^ Several ALK inhibitors have demonstrated potent anti‐tumour efficacy against *ALK*‐mutant NB, with most of these inhibitors entering early phase paediatric clinical trials.^18^ AURKA is a serine–threonine protein kinase known to stabilise the N‐Myc oncoprotein and promote cell growth specifically in *MYCN*‐amplified NB.^19^, ^20^ AURKA inhibitors have been demonstrated to be effective against *MYCN*‐amplified NB tumour models, via degradation of abundant N‐Myc oncoproteins, leading to apoptotic cell death.^21^ Finally, PI3K is a phospholipid kinase component of the PI3K/Akt/mTOR pathway, which is dysregulated in NB due to somatic mutations or overexpression.^22^ The efficacy of dual PI3K/mTOR inhibition has been widely observed in NB cell line models.^23^, ^24^, ^25^

Chemotherapy used in the treatment of NB has seldom changed over time, however recent genomic advances have allowed for targeted therapies to become more readily integrated into chemotherapy regimens to improve patient outcomes.^26^ Induction chemotherapy in NB patients involves the use of several chemotherapy agents in combination (e.g. carboplatin, cisplatin, cyclophosphamide, doxorubicin, vincristine and topotecan) to reduce primary tumour volume and metastatic potential.^27^, ^28^, ^29^ Following delayed surgical resection of primary tumours, maintenance therapies are then used to eliminate any minimal residual disease, and consist of; radiotherapy, cytokine immunotherapy and differentiating chemotherapy agents (e.g. 13‐*cis*‐retinoic acid). Therefore, identifying synergy between existing chemotherapy agents and novel targeted agents may improve clinical outcomes.

This study demonstrated DOT1L inhibitor SGC0946 combined with EZH2 inhibitor GSK343 had high selectivity against NB cells through induction of an ATF4‐mediated endoplasmic reticulum (ER) stress response. Moreover, the combination of SGC0946 and GSK343 significantly reduced tumour growth in comparison to single agent therapy, suggesting combined inhibition of EZH2 and DOT1L may represent an effective therapeutic approach for NB patients.

## 2 METHODS

### 2.1 Cell lines and tissue culture

The human NB cell lines: SK‐N‐BE(2)‐C (CVCL_0529, BE(2)‐C), SH‐EP (CVCL_0524), SK‐N‐FI (CVCL_1702I), LAN‐1 (CVCL_1827), SK‐N‐AS (CVCL_1700) and SH‐SY5Y (CVCL_0019) were maintained in Dulbecco's Modified Eagle Medium (DMEM) (#10566016 Gibco, Thermo Scientific, NSW, Australia) supplemented with 10% heat inactivated Foetal Bovine Serum (FBS) (Thermo Trace, Noble Park, VIC, Australia). KELLY (CVCL_2092) and CHP‐134 (CVCL_1124) human NB cell lines were maintained in Roswell Park Memorial Institute 1640 medium (RPMI) (#11875093 Gibco) supplemented with 10% heat inactivated FBS. The human fibroblast cell lines MRC‐5 (CVCL_0440) and WI‐38 (CVCL_0579) were cultured in Minimum Essential Medium alpha (MEMα) (#12571063 Gibco) supplemented with 10% heat inactivated FBS. SH‐EP, and SK‐N‐BE(2)‐C cell lines were kindly gifted from by Prof. June Biedler (Memorial Sloan Kettering Cancer Centre, New York, NY, USA). The LAN‐1 cell line was provided by Prof. John Maris. The KELLY cell line was kindly supplied by Dr Trevor Littlewood (Imperial Cancer Research Fund, London, UK). All other NB cell lines were purchased from the American Type Culture Collection (ATCC) (Manassas, VA, USA) or Sigma‐Aldrich (St. Louis, MO, USA). MRC‐5 and WI38 human fibroblast cell lines were purchased from ATCC. All cell lines used were authenticated using short tandem repeat (STR) profiling by Cell Bank Australia (Westmead, NSW, Australia) and were found to be free from mycoplasma contamination, following testing with a MycoAlert™ mycoplasma detection kit (#LT07‐118 Lonza, VIC, Australia) according to the manufacturer's instructions. All cell lines have been authenticated using short tandem repeat profiling within the last 3 years. All cell lines were incubated at 37°C with 5% CO_2_ and were passaged at 80%–90% confluency to maintain an exponential growth phase.

### 2.2 In vitro drug treatments

Following an initial 24 h of incubation after cell seeding, drug treatments were made in fresh DMEM or RPMI 1640 medium supplemented with 10% FBS. CDKI‐71 was kindly provided by Prof. Shudong Wang (University of South Australia: Adelaide, SA, Australia). CBL0137 was kindly provided by Dr Katerina Gurova (Department of Cell Stress Biology, Roswell Park Cancer Institute, Buffalo, USA). JQ1 (Cayman #11187), 13‐*cis*‐retinoic acid (13‐*cis*‐RA) (Sigma #R3255), SAHA (Cayman #10009929), GSK343 (Cayman #14094), SGC0946 (Cayman #13967), GSK‐LSD1 (Cayman #16439), 5‐AZA‐dC (Sigma #A3656), MLN8237 (Selleck #S1133), NVP‐BEZ235 (Cayman #10565), Crizotinib (Selleck #S1068) and Mafosfamide (Santa‐cruz #sc‐211761) were purchased commercially. Compounds were resolubilised in DMSO. Cells were incubated with compounds at 37°C with 5% CO_2_ for 6–72 h, followed by various phenotypic assays.

### 2.3 Cell viability/cytotoxicity assays

Cell viability was determined using a resazurin cell viability assay. 10X resazurin solution (75 mg resazurin (#R7017 Sigma‐Aldrich), 12.5 mg methylene blue (#M9140 Sigma‐Aldrich), 164.5 mg potassium hexacyanoferrate (III) (Sigma‐Aldrich), 211 mg potassium hexacyanoferrate (II) trihydrate (Sigma‐Aldrich) in 550 mL sterile PBS) was added to cells in cell culture medium seeded within 96‐well plates. Upon addition of the resazurin solution colorimetric plate readings were assessed using the Wallac 1420 VICTOR3™ microplate reader (PerkinElmer, USA), with excitation/emission wavelengths of 560/590 nm. A baseline reading for the plate was taken at 0 h following resazurin addition, followed by readings after 5–8 h of incubation at 37°C and 5% CO_2_. Cell viability was quantified as the percentage of vehicle control values.

### 2.4 High‐throughput combination drug screening

To identify possible combination therapies in NB, a high‐throughput combination drug screen was performed in the SK‐N‐BE(2)‐C cell line. SK‐N‐BE(2)‐C cells were seeded in 384‐well plates and allowed to incubate for 24 h at 37°C and 5% CO_2_, upon which cytotoxic compounds were added to plates using liquid dispensing robotics in the Drug Discovery Centre (Children's Cancer Institute, UNSW, Sydney, Australia). Plates were incubated at 37°C and 5% CO_2_ for 72 h prior to performing resazurin cell viability assays as described previously. In the screen, pairwise combinations of a total of 13 compounds (total of 78 unique combinations) were assessed using a 6 × 6 dilution matrix plate design for combinations of doses corresponding to ¼ × IC_50_, ½ × IC_50_, 1 × IC_50_, 2 × IC_50_ and 4 × IC_50_ for each drug, where possible (Table S1). The 13 selected agents covered multiple drug classes, either targeting epigenetic regulators (JQ1, SAHA, GSK343, SGC0946, GSK‐LSD1, 5‐AZA‐dC, CBL0137), deregulated kinases (MLN8237, NVP‐BEZ235, Critozinib, CDKI‐7), or clinically used agents in the disease (13‐*cis*‐RA, active cyclophosphamide metabolite, Mafosfamide). Following cell viability assays, data were normalised to the average value of all treatment controls on the plate and then converted to fractional effects (1 − (% cell viability)/100) for synergism calculations.

### 2.5 Synergism calculations for drug combinations

To determine relative synergy among the 78 drug combinations in the drug screen, normalised and averaged cell viability values were analysed using both Bliss's model of drug independence (BLISS)^30^ and the excess over the highest single agent model (HSA)^30^ to calculate synergism indices (SI). Previously calculated fractional effect values were subject to the BLISS model; wherein the SI is calculated by subtracting the sum and product of fractional effects from individual drugs (FE_drug1_ or _drug2_) from the fractional effect of the drug combination (FE_combo_) per dose combination.

$$\mathrm{SI}={\mathrm{FE}}_{\text{combo}}-\left({\mathrm{FE}}_{\text{drug1}}+{\mathrm{FE}}_{\text{drug2}}\right)-\left({\mathrm{FE}}_{\text{drug1}}\times {\mathrm{FE}}_{\text{drug2}}\right)$$

SI values for HSA are calculated by subtracting the fractional effect of the single drug with the highest activity (FE_HSA_) from the fractional effect of the combination treatment (FE_combo_) per dose combination.

$$\mathrm{SI}={\mathrm{FE}}_{\text{combo}}–{\mathrm{FE}}_{\mathrm{HSA}}$$

For all SI values, SI>0 is synergistic, SI = 0 is additive whilst SI<1 antagonistic. Averaged data sets were first calculated across doses using cell viability data from three independent experiments. The fractional effect (100 − % cell viability/100) was then determined for each dose and used as input to the CalcuSyn software along with the ratio of compounds used in each assay.^31^ SGC0946 was combined with GSK343 at a constant ratio of 1:1. Correlation coefficients (r) were used to confirm the strength of the dose‐effect relationships in CalcuSyn, wherein *r* > 0.95 represented strong correlations.

### 2.6 RNA isolation, cDNA synthesis, qPCR and microarray profiling

RNA isolation from cell lines was performed using the PureLink™ RNA Mini Kit (#12183018A Invitrogen) according to the manufacturer's instructions. cDNA synthesis was performed using the Bioline kit according to the manufacturer's instruction. qPCR was conducted using SYBR Green on the QuantStudio 3 instrument using several primer sequences (Table S2). SK‐N‐BE(2)‐C and KELLY cells were seeded in T25 flasks. Following 24 h of incubation cell culture medium was replaced with medium containing either DMSO, 12.8 μM SGC0946, 12.8 μM 93 GSK343 or a combination of both drugs. Treated cells were incubated for 6 h at 37°C and 5% CO_2_, followed by total RNA extraction, quantitation and quality control as described above. RNA from treated samples were then subject to microarray profiling via the PrimeView Human Genome U219 Array (#901605 Applied Biosystems) according to manufacturer instructions, by the Ramaciotti Centre for Genomics (UNSW, Australia).

### 2.7 Microarray data analyses

Array CEL files, containing the raw probe intensities measured from the microarray, were imported into R using the oligo R package.^32^ Raw probe intensity values were then subject to robust multi‐array averaging (RMA) normalisation using the oligo package. PrimeView probes were annotated with ‘hgu219.db’ package annotations alongside the biomaRt package.^33^ Genes with multiple microarray probes were collapsed using interquartile range with a custom R script, which removed probes with lower intensities.^34^ Differential gene expression between each set of conditions were then calculated using linear regression models in the limma R package,^35^ *p*‐values were corrected for multiple comparisons using the Benjamini‐Hochberg method. Differentially expressed genes were those genes with a log2‐transformed fold change >0.5 and adjusted *p* < 0.05. Pathway enrichment among differentially expressed genes between treatment conditions were determined by using the clusterProfiler R package with ReactomePA and GO databases.^36^

### 2.8 Protein extraction, quantitation, SDS‐PAGE and western blot

Cells were lysed in radio‐immunoprecipitation assay (RIPA) lysis buffer (#89901 Thermo Scientific) with protease inhibitor cocktail (#P2714 Sigma‐Aldrich). The total cell lysate was quantified using the Pierce BCA (bicinchronic acid) Protein Analysis Kit (#23227 Thermo Scientific) according to the manufacturer's instructions. Assay absorbance was measured using the VICTOR3™ microplate reader at an absorbance wavelength of 570 nm. Thirty microgram of cell lysate was reconstituted in SDS loading buffer with reducing agent (Bio‐Rad). Samples were denatured at 95°C for 10 mins. SDS‐PAGE was then performed by loading samples into a 12 or 18‐well 10.5%–14% Tris–HCl precast Criterion Gel (#3459949 or #3459950 Bio‐Rad) residing in a Criterion™ running cell (Bio‐Rad) containing Tris‐Glycine‐SDS (TGS) running buffer (10 × TGS; 30 g Tris‐Cl, 150 g Glycine, 10 g SDS in 1 L MilliQ H2O). Precision Plus Protein™ Dual Colour Standards (#1610374 Bio‐Rad) were also loaded. Proteins were then transferred from the gel to a 0.45 μm supported nitrocellulose membrane (GE Healthcare, Rydalmere, NSW, Australia). Protein transfer was carried out in a Criterion blotting cell (Bio‐Rad) containing transfer buffer (TB) (10 × TB; 30 g Tris‐Cl, 112 g Glycine in 1 L MilliQ H_2_O). Protein transfer was confirmed using Ponceau S staining buffer (0.2 g Ponceau S [#P7170, Sigma‐Aldrich] in 5 mL glacial acetic acid and 94.8 mL MilliQ H_2_O).

Membranes underwent blocking for 2 h at room temperature in 10% nonfat skim milk powder in Tris‐Buffered Saline (TBS) (10 × TBS; 24 g Tris‐Cl, 88 g NaCl in 1 L MilliQ H2O) + 1% Tween 20 (TBST). Membranes were washed in TBST and incubated overnight at 4°C in primary antibody dilutions constituted with 0.5% nonfat skim milk powder in TBST using an orbital shaker. These dilutions contained primary antibodies against human: N‐Myc (#sc‐791 Santa‐Cruz Biotechnology, 1:5000), Vinculin (#ab130007 Abcam, 1:50000), GAPDH (#MA1‐16757 Invitrogen, 1:1000), EZH2 (CST #5246, 1:1000), DOT1L (CST #77087, 1:1000), H3K27me3 (CST #9733, 1:1000) and H3K79me2 (CST #5427, 1:1000) at varied dilutions. Vinculin and GAPDH were utilised as loading or housekeeping controls. Membranes were washed in TBST and incubated in secondary antibody dilutions in 0.5% nonfat skim milk powder in TBST at room temperature for 2 h using rabbit anti‐goat or mouse anti‐goat horseradish peroxidase antibodies (Thermo Scientific Surrey Hills, VIC, Australia, 1:2000). Membranes were washed in TBST and then incubated with SuperSignal™ West Pico Chemiluminescence Substrate (#34079 Thermo Scientific). The ChemiDoc™ Touch Imaging System (Bio‐Rad), was used to detect chemiluminescent signals from the membranes. Densitometry was conducted using the Quantity One software (Bio‐Rad). All samples were normalised to their respective loading control and then to experimental controls.

### 2.9 Flow cytometry

For flow cytometry assays SK‐N‐BE(2)‐C and KELLY NB cell lines were seeded in 6‐well plates. Cell culture medium was replenished with fresh medium containing either DMSO, GSK343, SGC0946 or a combination of agents. After 24 h, cell culture medium was aspirated, and cells were harvested via trypsin dissociation. To detect apoptotic cell death in treated cells, cells were stained with the PE Annexin V Apoptosis Detection (#559763 BD Biosciences) according to manufacturer instructions. The cells were resuspended in 1 × binding buffer and stained with 5 μL Annexin V‐PE conjugated antibody and 5 μL 7‐AAD, where cells were vortexed and incubated at room temperature in the dark for 15 mins. After staining, 400 μL of 1 × binding buffer was added to each tube and cells were analysed via flow cytometry. Annexin V is present on the surface of apoptotic cells and 7‐AAD indicates whether the cell membrane is intact. When used together these markers can highlight cells in either early (Annexin V+/7‐AAD‐) or late apoptosis (Annexin V+/7‐AAD+) as well as live (Annexin V−/7‐AAD‐) and necrotic cells (Annexin V−/7‐AAD+). Unstained and single positive staining controls were established, where 5 μM Camptothecin supplied with the kit was used to induce apoptosis and cell death as a positive control.

### 2.10 Metabolomic profiling of cells

SK‐N‐BE(2)‐C cells were seeded in T25 flasks. The following day, culture medium was replenished with DMEM containing either; DMSO (control), 12.8 μM SGC0946, 12.8 μM GSK343 or a combination of both drugs. Cells were left to incubate for 6 h at 37°C, upon which the medium was aspirated, and cells were washed with chilled PBS 2 × while still attached to the flask. Following cell scraping, cells were counted using 0.4% Trypan Blue. After centrifugation at 300 × g for 3 mins, cells were resuspended in metabolite extraction buffer (50% methanol [#322415 Sigma‐Aldrich], 30% acetonitrile [#34851 Sigma‐Aldrich], 20% milliQ H_2_O) followed by incubation in a thermo‐mixer at 4°C and 1400 rpm for 15 mins. Following extraction cells were centrifuged at 4°C and 16,000 × g for 10 mins, where the resulting supernatant was carefully transferred into HPLC vials (Sigma‐Aldrich) and sealed. Samples were then profiled using hydrophilic interaction chromatography mass spectrometry (HILIC‐MS) through a Q Exactive™ Plus Hybrid Quadrupole‐Orbitrap™ Mass Spectrometer (ThermoFisher Scientific, Waltham, MA, USA) at the Bioanalytical Mass Spectrometry Facility (BMSF) (Mark Wainwright Analytical Centre, UNSW, NSW, Australia). Mass spectra were acquired and processed using Xcalibur™ software (version 3.0, ThermoFisher Scientific), and the resultant data were further processed to annotate metabolites through the Compound Discoverer™ software (version 3.0, ThermoFisher Scientific). Resultant mass spectra areas, which summarise the relative abundance of each metabolite, were normalised to the cell numbers used for input into the assay to account for technical artefacts arising from cell loss during metabolite extraction. For all comparisons paired t‐tests were utilised, followed by adjustments of *p*‐values with the Benjamini‐Hochberg method to account for multiple comparisons between treatment groups and then to comparisons made with all other metabolites. For these analyses metabolites with a raw *p* < 0.05 and adjusted *p* < 0.1 were considered to be significant.

### 2.11 NB cell line xenograft models

For determining the maximum tolerated dose of SGC0946 and GSK343, the compounds were administered to 5‐week‐old immunodeficient Balb/c‐Fox1nu/Ausb (Balb/c nude) mice using a dose escalation strategy. Balb/c nude mice sourced from ABR (Mossvale, NSW, Australia) were grouped into either SGC0946, GSK343 and combination cohorts. Each cohort consisted of a control group which were respectively, 5% DMSO in saline (0.9% NaCl), 5% EtOH in saline (0.9% NaCl) and 5% DMSO +5% EtOH in saline (0.9% NaCl). Within each treatment cohort there were several treatment groups in which mice were treated with 7.5, 15, 30 and 60 mg/kg/day of the specified agent via intraperitoneal injections using 27G needles (BD Biosciences) on 5 days on, 2 days off schedule, where injection sites were alternated each day. Mice were monitored daily, which included the measurement of body weight, assessment of physical condition and behavioural characteristics following drug treatment. The treatment period lasted 21 days, at the end of which mice were humanely killed by CO_2_ asphyxiation and organ tissues were collected and weighed. If mice displayed any signs of weight loss >20% of their previous highest body weight or any degradation of physical or behavioural well‐being, then mice were humanely killed by CO_2_ asphyxiation and organ tissues were collected for histological analyses.

For assessing anti‐tumour efficacy of the compounds, 5 × 10^6^ SK‐N‐BE(2)‐C cells were subcutaneously injected, using an 27G needle, into the right flank of Balb/c nude mice sourced from ABR (Mossvale, NSW, Australia). Tumours were monitored daily and measured using Kincrome™ vernier digital callipers with the calculation; 0.5 × length (mm) × (width (mm)).^2^ When tumours reached a volume of 50 mm^3^, mice were assigned into treatment groups of either; 5% DMSO and 5% EtOH in saline (0.9% NaCl), 30 mg/kg/day SGC0946, 30 mg/kg/day GSK343 or a combination of the drugs. Mice were treated by intraperitoneal injections using 27G needles on a 5 day on, 2 days off schedule where injection sites were alternated each day. Treatments lasted for up to a total period of 42 days or until tumours reached a volume of 1000 mm^3^, at which point, the mice were humanely killed by CO_2_ asphyxiation and tumour tissues collected for subsequent histological and protein analyses. Xenograft studies were both granted ethical consent by the UNSW Animal Care and Ethics Committee (ACEC 19/88B).

### 2.12 Software and statistics

For those cytotoxicity assays assessing dose responses across a range of drug concentrations, the IC_50_ was determined by fitting non‐linear regression curves (Sigmoidal dose–response—variable slope) to normalised dose–response data, where the upper limit of the data was constrained to 100, using the GraphPad Prism 7 software. Flow cytometry data was analysed using the FlowJo software (FlowJo version 10.6.1) (Ashland, OR, USA). Cells were first gated based on front‐scatter and side‐scatter parameters to eliminate cell debris, and then based on unstained negative controls and positive controls where possible.

## 3 RESULTS

### 3.1 Combinatorial drug screening reveals strong anticancer synergy between histone methyltransferase inhibitors

To identify novel targeted combination therapies in NB, we performed a high‐throughput combinatorial drug screen in the chemo‐resistant cell line SK‐N‐BE(2)‐C, derived from a NB patient tumour after chemotherapy, with *MYCN* gene amplification and mutant TP53, markers of highly aggressive and drug‐resistant disease. In this screen, 78 pairwise combinations of 13 compounds were tested, by analysing cell viability matrices following drug treatment of SK‐N‐BE(2)‐C cells in vitro across a range of doses flanking the IC_50_ for each drug. The chosen drugs targeted epigenetic regulators (JQ1, SAHA, GSK343, SGC0946, GSK‐LSD1, 5‐AZA‐dC, CBL0137)^11^, ^37^, ^38^, ^39^, ^40^, ^41^ deregulated kinases known as driver genes in NB (MLN8237, NVP‐BEZ235, Crizotinib, CDKI‐71)^42^, ^43^, ^44^, ^45^ and, clinically used agents in the disease (13‐*cis*‐RA, Mafosfamide)^46^, ^47^ (Table S1 and Figure S1A,B).

**FIGURE 1:**
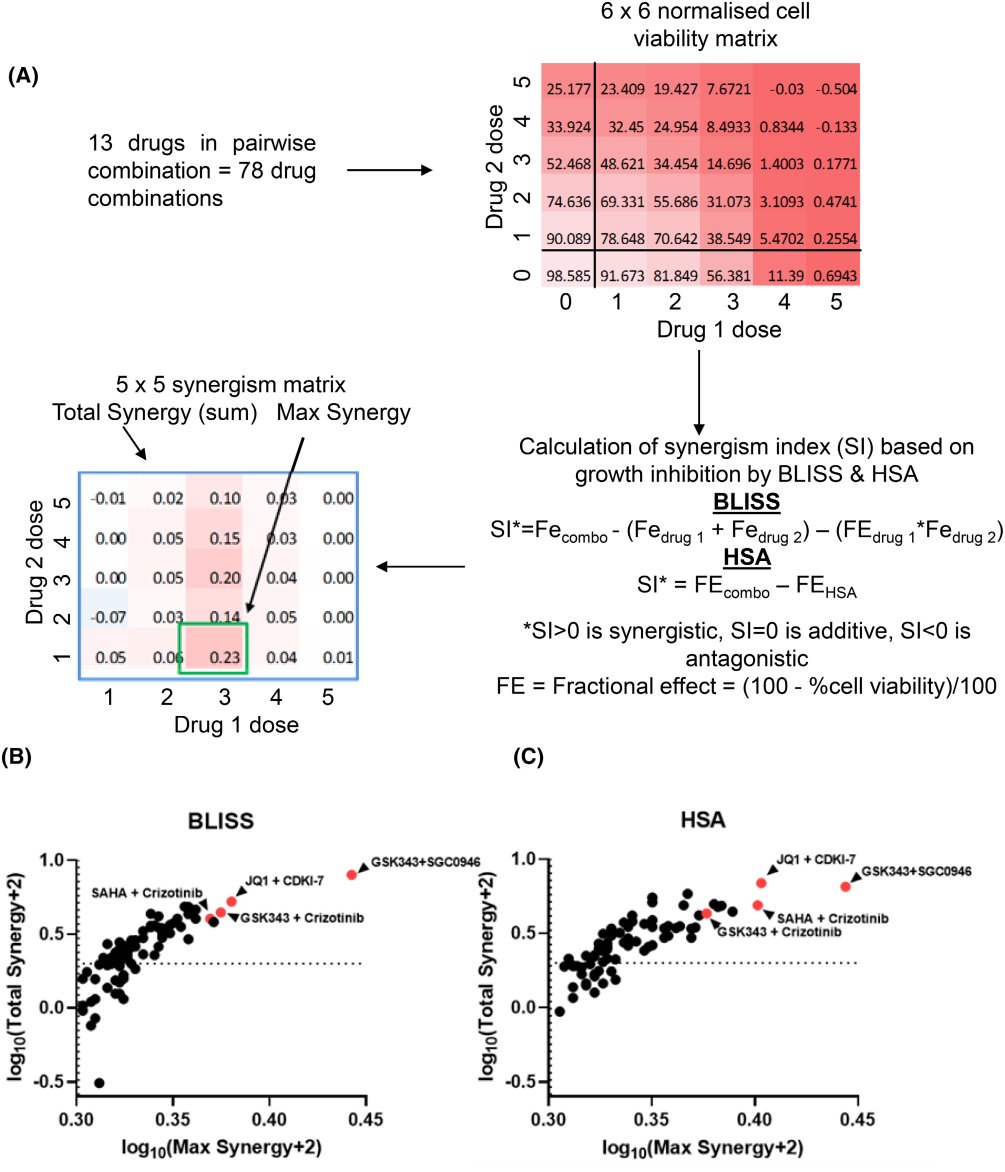
Drug combination screening identifies highly synergistic drug combinations against the SK‐N‐BE(2)‐C neuroblastoma cell line. (A) Schematic of the drug combination screen and downstream data analysis. The treatment control (DMSO) normalised cell viability data are subject to BLISS and HSA models to calculate synergism indices (SI). First the fractional effect (FE) is calculated from normalised cell viability values. Then FE values are subject to BLISS; wherein SI is calculated by subtracting the sum of FE and multiple of FE of individual drugs (FE_drug1/drug2_) from the FE of the drug combination (FE_combo_) per dose combination. SI values for HSA are calculated by subtracting the FE of the single drug with the highest activity (FE_HSA_) from FE_combo_. For all SI values, SI >0 is synergistic, SI = 0 is additive whilst SI <0 is antagonistic for that specific dose pair. Finally, the total synergy for the drug pair is calculated by taking the sum of the 5 × 5 synergism matrix, and the max synergy for the drug pair is the highest value from the synergism matrix. (B) BLISS and (C) HSA synergism summary metrics are plotted in a scatterplot to illustrate synergism of drug pairs. The x‐axis represents the maximum synergy and y‐axis represents total synergism within the synergism matrix of each drug pair. These values were transformed into the log‐space using log_10_(×+2). Synergism thresholds are indicated by dotted lines (SI = 0), values above these lines are synergistic. Drug pairs which were highly synergistic across both models are highlighted in red.

Following pairwise combinatorial drug screening, cell viability matrices were normalised to treatment controls and then analysed using both Bliss's model of drug independence (BLISS)^48^ and the excess over the highest single agent model (HSA)^49^ to determine the relative synergism among the 78 drug combinations (Figure 1A). In order to assess synergy across all dose combinations the ‘total synergy’ of the drug pair was then calculated as the sum of all synergism values in each matrix and the ‘maximum synergy’ of the drug pair was calculated as the highest synergism value in the matrix for both BLISS and HSA models across each pairwise combination of drugs (Figure 1A, Table S3).

After analysing the ‘maximum’ and ‘total’ synergy of the 78 drug combinations, top hits were classified by identifying highly synergistic combinations across both BLISS and HSA methods (Figure 1B,C). The highest synergism was observed between two HMT inhibitors, GSK343 and SGC0946.

### 3.2 SGC0946 combined with GSK343 has high selectivity against NB cells

**FIGURE 2:**
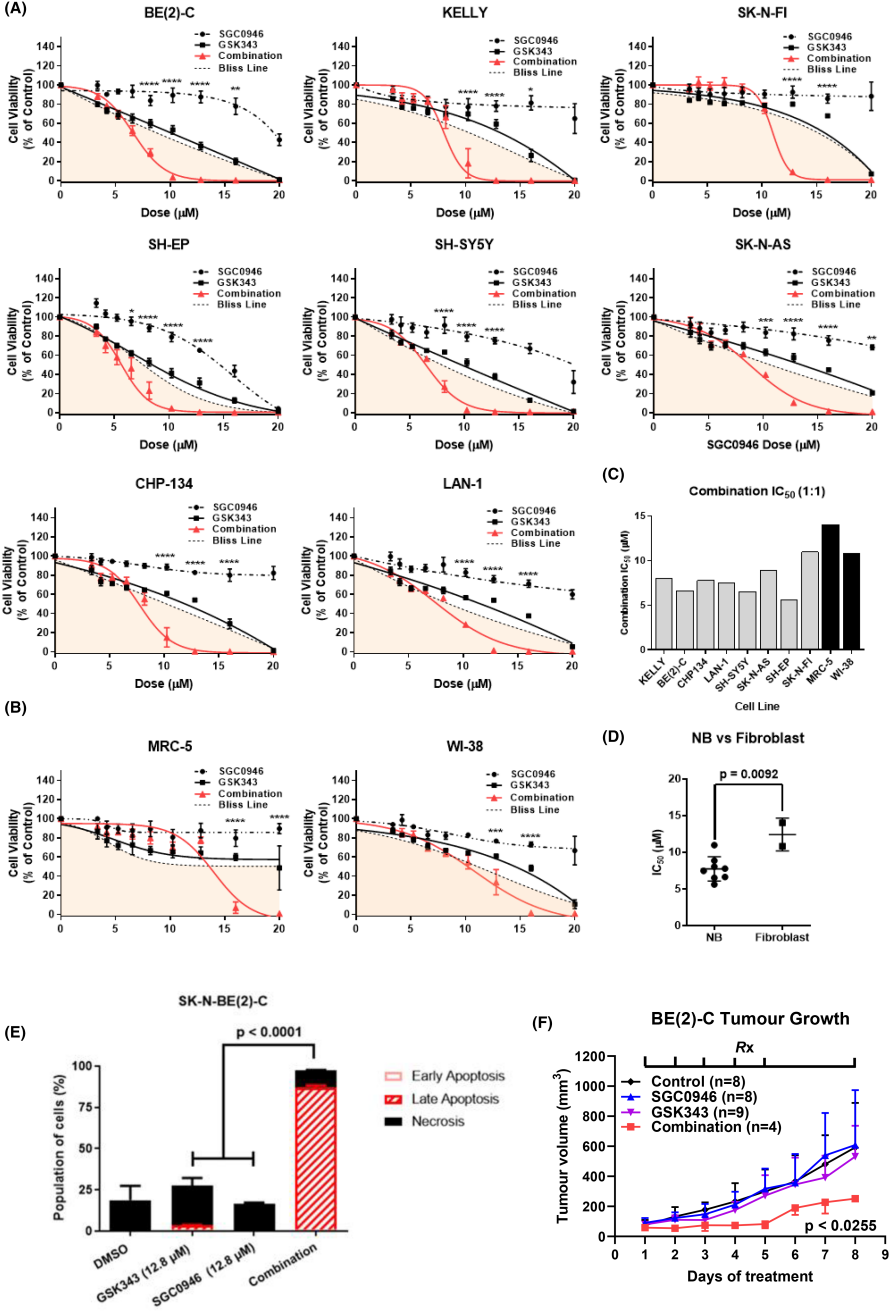
SGC0946 combined with GSK343 has high selectivity against neuroblastoma cells. (A) Resazurin cell viability assays were performed on a panel of 8 neuroblastoma (NB) cell lines (SK‐N‐BE(2)‐C, CHP134, KELLY, SH‐SY5Y, LAN‐1, SK‐N‐AS, SH‐EP and SK‐N‐FI) 72 h posttreatment using an expanded dose range (9‐point) for SGC0946, GSK343 or the combination of these drugs in a 1:1 ratio. (B) The same cell viability assays were performed in human fibroblast cell lines (MRC‐5, WI‐38). Error bars represent the standard error of the mean for at least three independent experiments. For each plot a ‘Bliss Line’ is provided, which represents the expected result if both compounds were additive in nature, and values below the line in the shaded region indicate synergistic dose combinations. Furthermore, *p*‐values are reported for significant differences between the combination and both single‐agents using two‐way anova analyses (*p < 0.05, **p < 0.01, ***p < 0.001, ****p < 0.0001). (C) IC_50_'s for the combination of drugs are presented in a column graph for the eight neuroblastoma and two fibroblast cell lines. (D) IC_50_'s is grouped into neuroblastoma (NB) cell lines or fibroblast categories. Reported *p*‐value is from a two‐sided unpaired *t*‐test between the two groups. Error bars represent the standard error of the mean IC_50_'s for each group. (E) Apoptotic and necrotic effects of the single agents and combination treatment were determined following pre‐treatment of SK‐N‐BE(2)‐C cells for 24 h before collecting the cells and staining with Annexin V/7‐AAD. Staining was detected by FACS analysis. A summary of results is shown as the percentage of cells in early or late apoptosis, and necrosis as histograms. Significance was determined from three independent experiments. (F) Tumour growth curves of Balb/c nude mice, xenografted with SK‐N‐BE(2)‐C cells, following treatment with either a vehicle control, 30 mg/kg/day SGC0946, 30 mg/kg/day GSK343 or the combination of these treatments. The treatment schedule (Rx) is indicated on the top of the plot, wherein drugs were administered on a 5 day on and 2 days off schedule with intraperitoneal injections. Averaged tumour growth curves are shown for time points where all mice in each treatment arm were alive. The reported *p*‐value is from the comparison between combination and single agent/control treatments derived from a one‐way anova with mixed‐effects analysis, corrected with Tukey's multiple comparison test. Error bars represent the standard error of the mean and are one‐sided for visualisation.

Given the promising synergy of GSK343 with SGC0946, further cell viability dose response assays were designed to validate synergism and assess therapeutic windows in a panel of NB and normal fibroblast cell lines. Synergism between the compounds was clearly observed in all NB cell lines from 8.2 to 20 μM (1:1 ratio between GSK343 and SGC0946) using the BLISS synergism model (Figure 2A). Given the array of molecular features present in this panel of cell lines, synergy is therefore independent of any known molecular alterations. Synergy was also observed in human fibroblast cell lines albeit at much higher doses compared with NB cell lines from 16 to 20 μM (Figure 2B). Taking the average BLISS scores across all 9 doses for each cell line revealed overall synergism (BLISS >0) in NB cell lines compared with additivity/antagonism in fibroblast cell lines (BLISS <0) (*p* = 0.0003) (Table S4). These findings suggested that the mode of synergy for these compounds was more selective for NB cells compared with normal fibroblasts.

Synergy was additionally calculated using the Chou‐Talalay method to generate combination indices (CI) at the IC_50/75/90_ for the combination.^31^ Generally, NB cell lines displayed weak synergy at the IC_50_ (0.9 < CI <1), had moderate synergy at IC_75_ (0.9 < CI <0.4) and had the strongest synergy (0.7 < CI <0.2) at the IC_90_ dose with the exception of the SH‐EP cell line which displayed weak synergism throughout (CI > 0.9) (Table S4).

Among the different NB cell lines, we found similar IC_50_ concentrations for the HMT inhibitor combination which suggested that the diverse molecular features of each NB cell line did not influence sensitivity to the drug combination (Figure 2C), thereby representing a clinical benefit in the context of complex and evolving molecular features in relapsed NB patients.^50^ Additionally, when comparing the IC_50_ of the combination (1:1 ratio) for NB cell lines to that of normal fibroblasts we found NB cell lines were 1.6‐fold more sensitive (Figure 2D). To investigate whether the combination induced apoptosis of NB cells, SK‐N‐BE(2)‐C cells were treated with either DMSO control, GSK343, SGC0946, or the combination (1:1 ratio) for 24 h. Cells were then stained with Annexin V/7AAD and assessed by flow cytometry. The percentage of cells which had undergone late apoptosis was 2.07% for GSK343 and 0.037% for SGC0946 monotherapy compared with 87.7% for the combination (Figure 2E and Figure S2A). We next assessed the toxicity and anti‐tumour efficacy of these compounds using xenografted SK‐N‐BE(2)‐C cells in the flank of immune‐deficient Balb/c nude mice. After establishing the maximum tolerated dose of SGC0946 and GSK343 in Balb/c nude mice (Figure S2B,C), we assessed the efficacy of the combination in vivo against NB tumour xenografts. SK‐N‐BE(2)‐C cells were xenografted into the flank of immunodeficient Balb/c nude mice and tumours were allowed to grow to 50 mm^3^ in volume prior to therapy initiation. SGC0946 and GSK343 were administered as previously described at 30 mg/kg/day,^51^, ^52^ along with single agent and vehicle control arms for up to 3 weeks. Tumour volumes were assessed daily, and the assay endpoint was set at a tumour volume of 1000 mm^3^. All tumours reached the 1000 mm^3^ endpoint by 26 days, suggesting that these treatments could not achieve complete tumour regression and/or remission. However, by observing tumour growth when all mice were still alive (Days 1–8), the combination therapy was able to significantly reduce tumour growth compared to all other treatment arms and vehicle control (Figure 2F).

### 3.3 High expression of both *EZH2* and *DOT1L* best predicts poor NB patient outcome

We used the publically available data from two (Kocak^53^ and SEQC^54^) large NB patient primary tumour total mRNA databases to confirm the findings of previous studies that high expression of either *EZH2* or *DOT1L*, dichotomised around their median expression level, correlated with poor NB patient prognosis (Figure S3A–D).^11^, ^55^, ^56^, ^57^ Cox proportional hazard (CoxPH) modelling was performed to determine whether *EZH2* or *DOT1L* expression could predict poor outcome in NB patients, both as single variables and when considered together with other known prognostic factors in the disease.^58^ High expression of either *EZH2* or *DOT1L* predicted poor NB patient outcome, as determined by hazard ratios between 1.4 and 3.2 for EFS/OS across both Kocak and SEQC cohorts (Table S5). Moreover, high *EZH2* or *DOT1L* expression had prognostic significance independent of *MYCN* amplification status, age of diagnosis and International Neuroblastoma Staging System (INSS) disease stage.

**FIGURE 3:**
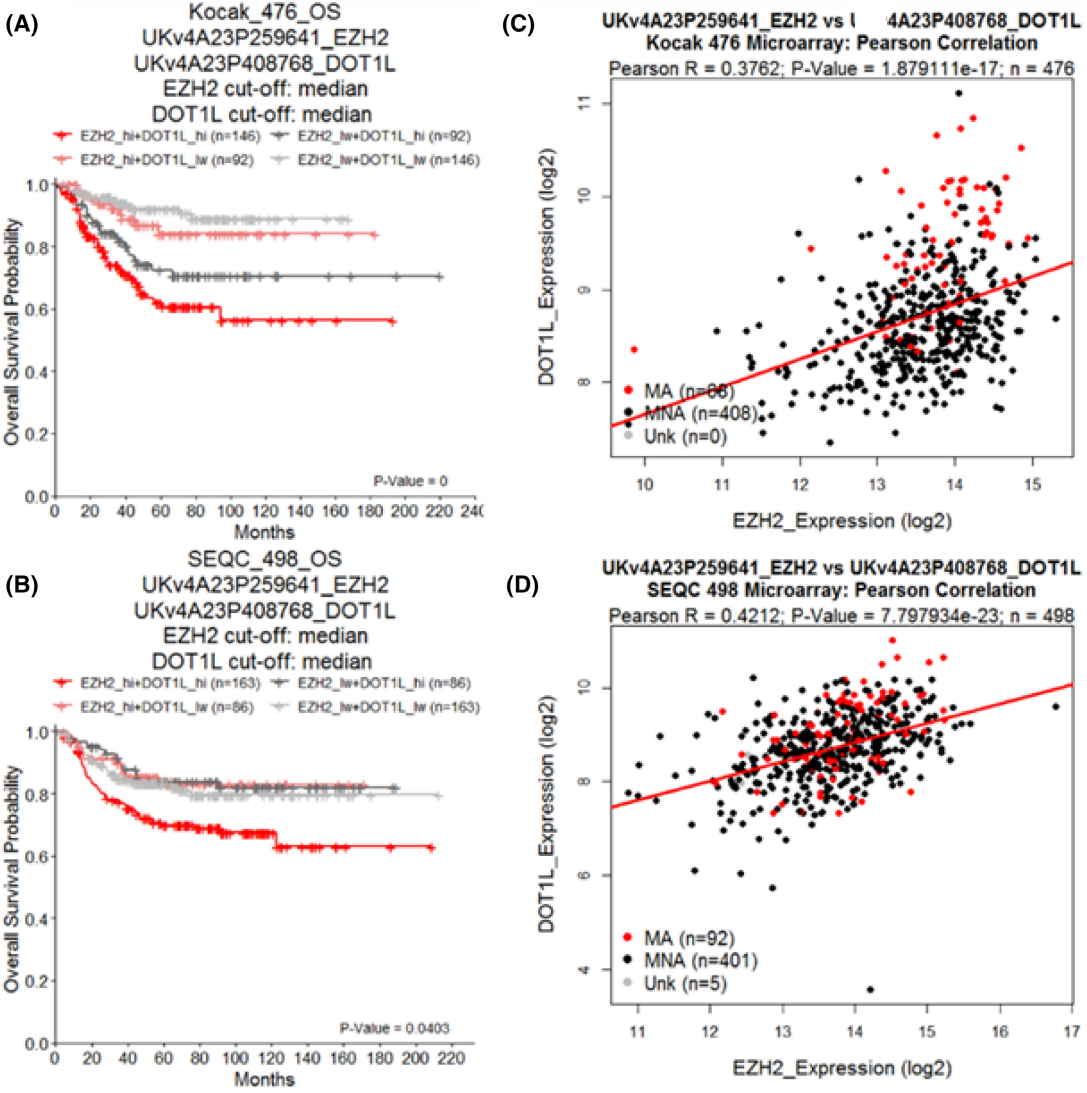
High expression of both EZH2 and DOT1L predicts poor neuroblastoma patient outcome. Kaplan–Meier curves of overall survival (OS) of neuroblastoma patient cohorts subdivided by the median expression of either EZH2 or DOT1L. Cohorts are annotated at the top of each plot (Kocak, SEQC), as well as the microarray probe ID used for the given gene. The reported *p*‐value is from a log‐rank test comparing the two curves. Cohorts were further subdivided using both median EZH2 and DOT1L expression to create four sub‐groups (EZH2 high + DOT1L high, EZH2 high + DOT1L low, EZH2 low + DOT1L high, EZH2 low + DOT1L low). These sub‐groups were classified in either (A) Kocak or (B) SEQC NB neuroblastoma patient cohorts and plotted as Kaplan–Meier curves with respect to EFS or OS survival times. The reported BH adjusted *p*‐value represents the log‐rank comparison between the EZH2+DOT1L high subgroup and the EZH2+DOT1L low subgroup after correcting for multiple comparisons with other groups. Pearson correlations between EZH2 and DOT1L gene expression were made in (C) Kocak and (D) SEQC cohorts. Pearson correlation coefficients (r) are provided at the top of each plot, along with associated *p*‐values and sample sizes (n). Dots are coloured by MYCN amplification status, either being amplified (MA, red), non‐amplified (MNA, black) or unknown (Unk, grey).

We found that patients who had high expression of both *EZH2* and *DOT1L* had the poorest prognosis in both Kocak and SEQC cohorts (Figure 3A,B). This suggested that there was potential oncogenic cooperation between the two genes in driving the disease. The expression of *EZH2* and *DOT1L* were further assessed for correlations across patient tumours from the two large databases. *EZH2* was found to be significantly and positively correlated (R2 0.3762 and 0.4212/p 1.87e‐17 and 7.80e‐23) with *DOT1L* gene expression (Figure 3C,D). Our findings suggest that SGC0946 and GSK343 combination therapy may be most effective in those patients expressing both *EZH2* and *DOT1L* highly, and thus could be used as a biomarker in future experimental trials.

### 3.4 Combination therapy induces an ER stress response expression profile in NB cells

**FIGURE 4:**
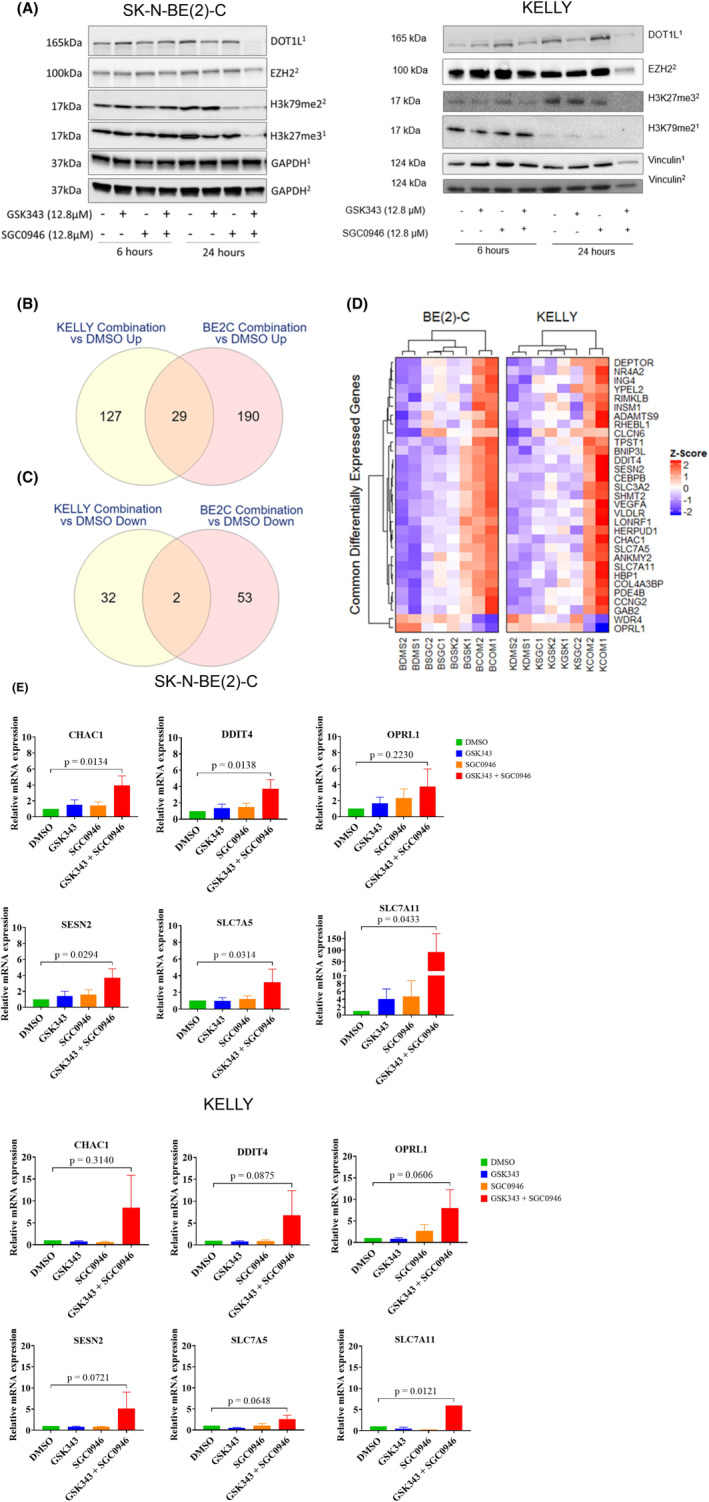
Combination therapy induces an ER stress response expression profile in NB cells. (A) Western blot analyses of cell lysates from treated cells for DOT1L, EZH2, H3K27me3, H3K79me2, Vinculin and GAPDH proteins. Cells were treated with a DMSO vehicle control or 12.8 μM of drug for 6 or 24 h followed by protein extraction. The size at which bands were detected (kDa) are shown to the left of the plot and respective treatment conditions/time points are shown below the plot. Specific antibodies were probed on different blots, where superscripts 1 and 2 annotated on the right of the plot indicates matching blots. (B, C) Venn diagrams depict the number of common differentially expressed genes (B) upregulated or (C) down‐regulated by the combination treatment in both SK‐N‐BE(2)‐C and KELLY cells. (D) Scaled gene expression heatmap of 31 common differentially expressed genes between SK‐N‐BE(2)‐C (B) and KELLY (K) cells after combination treatment. Columns represent individual replicates of the conditions (DMS = DMSO Control, SGC=SGC0946, GSK = GSK343, COM = Combination) and rows represent commonly regulated genes, both of which were hierarchically clustered. Gene expression scale (z‐score) is provided to the right of the heatmap. (E) qRT‐PCR assessing the mRNA expression levels of the top six ER stress target genes CHAC1, DDIT4, OPRL1, SESN2, SLC7A5 and SLC7A11 in SK‐N‐BE(2)‐C and KELLY cells from the microarray. The cells were treated with DMSO control, GSK343 (12.8 μM), SGC0946 (12.8 μM) and with combination of GSK343 and SGC0946 (1:1) for 6 h. For relative quantification of mRNA expression, expression levels of cells treated with DMSO were set to 1. Bars depict mean values ± standard error of the mean (SEM) of three different experiments. PCR reactions were performed in triplicates.

To investigate potential mechanisms of drug synergy, RNA and protein were extracted from SK‐N‐BE(2)‐C and KELLY cell lines following treatment with either the DMSO control, 12.8 μM SGC0946, 12.8 μM GSK343 or the combination of both drugs for a period of 6 and 24 h, which had reflected the most synergistic doses in the 72 h cytotoxicity assays. The 6 h time point was chosen in order to capture early changes in mRNA after treatment. SGC0946 and GSK343 canonically target DOT1L and EZH2 HMTs respectively, leading to altered H3K27me3‐ and H3K79me2‐mediated transcriptional changes. We first confirmed that the targets of these HMT inhibitors were being reduced through Western blot analyses. Here we observed reductions in the target HMT inhibitors and protein levels of H3K79me2 and H3K27me3 at 24 h post combination treatment (Figure 4A). Differentially expressed genes were defined as having a log_2_‐transformed fold change (between each treatment and control) >0.5 or <−0.5 and having a Benjamini‐Hochberg (BH) adjusted *p* < 0.05. In SK‐N‐BE(2)‐C cells, single agents did not induce any gene expression changes beyond our significance threshold after 6 h. However, when treated with the combination therapy, 55 genes were downregulated and 219 upregulated (Figure S4A). In KELLY cells, SGC0946 monotherapy significantly induced 16 genes, GSK343 upregulated 4 genes and downregulated 1, whereas the combination induced 156 genes and downregulated 34 (Figure S4B). Taken together, the combination of drugs caused a far higher number of gene expression alterations compared to single agents, implying that the combination operated via a unique mechanism to impart cytotoxicity, compared to single agents.

A total of 31 differentially expressed genes (29 upregulated, 2 downregulated) were found to be altered by the combination treatment in both cell lines (Figure 4B–D and Table S6). Several ER stress‐related genes were upregulated by the combination in both cell lines: *CHAC1*, *DDIT4*, *OPRL1*, *SESN2*, *SLC7A5* and *SLC7A11*.^59^, ^60^, ^61^, ^62^, ^63^, ^64^, ^65^ Quantitative real time qPCR analyses showed that mRNA expression levels of all 6 candidate genes were strongly induced with a fold change range of 1.6–14.1 in SK‐N‐BE(2)‐C and a fold change range of 2.1–6.6 in KELLY cells after 6 h of drug treatment with the combination, compared to controls (Figure 4E).

**FIGURE 5:**
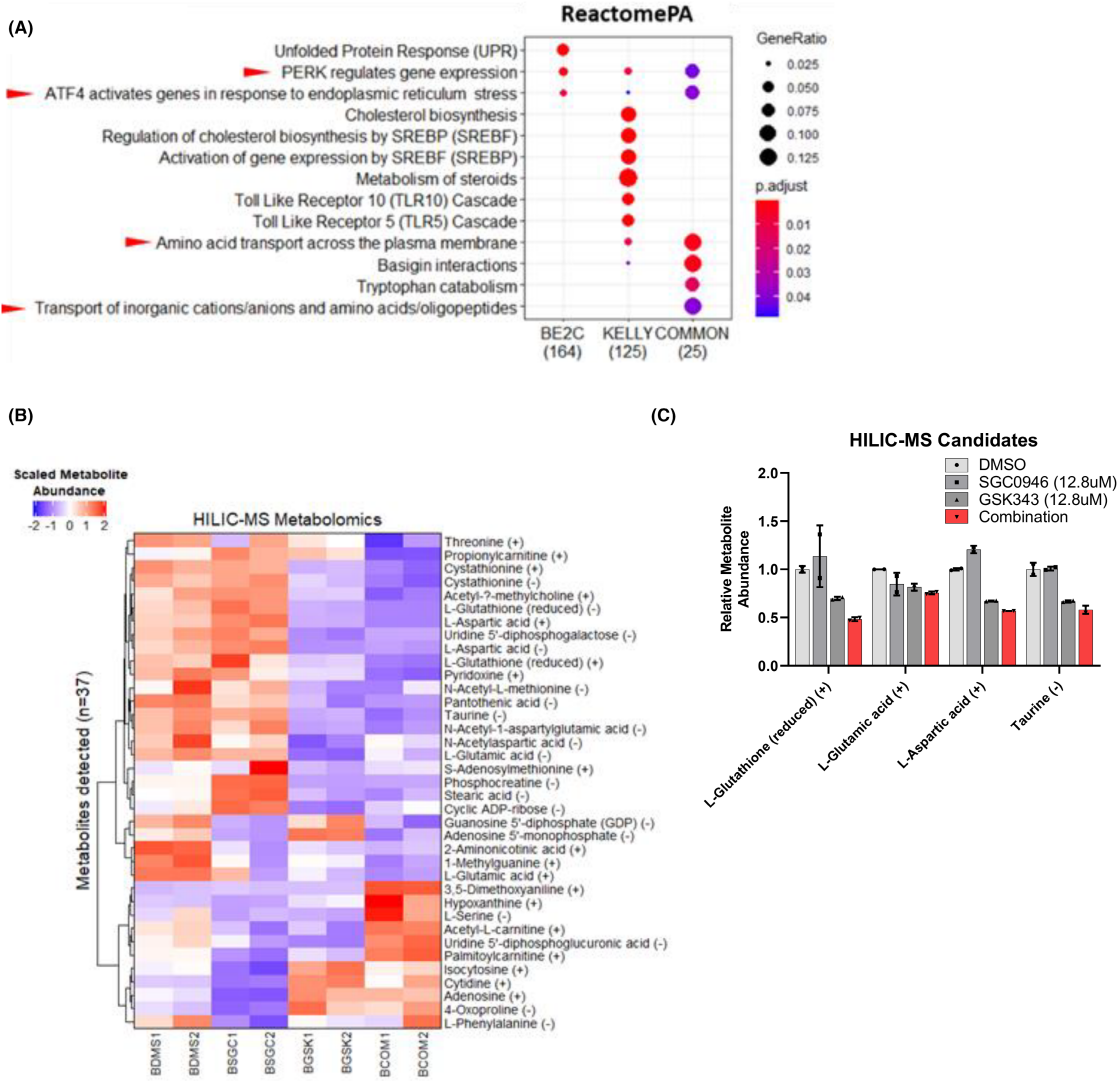
Gene set analyses in combination treated SK‐N‐BE(2)‐C and KELLY cells reveals an ATF4‐driven ER stress response. (A) Over‐representation analyses (ORA) using differentially expressed genes in combination treated KELLY or SK‐N‐BE(2)‐C cells versus control were performed using the ReactomePA database. The ‘COMMON’ differentially expressed genes previously identified were also used as input for each analysis. The results are represented as enrichment dot plots, wherein the size of the dot represents the gene ratio (number of genes in gene set/total number of genes in gene set) and the colour of the dot represents the Benjamini‐Hochberg adjusted *p*‐value after a Fisher's exact test for each pathway. The number of differentially expressed genes in the presented gene sets are also indicated below each plot. Only significantly over‐represented gene sets are shown (adjusted *p* < 0.05). Red arrows indicate either ATF4, ER stress or amino acid related gene sets. (B) Glutathione (GSH) and several amino acids are depleted by the combination of SGC0946 and GSK343. SK‐N‐BE(2)‐C cells were treated for 6 h followed by metabolomic profiling using hydrophilic interaction chromatography mass spectrometry (HILIC‐MS). Heatmap of HILIC‐MS detected metabolites, where rows represent metabolites (*n* = 37) and the columns represent samples. A scale bar is provided at the top left of the plot, indicating scaled metabolite abundance. HILIC separates positively (+) and negatively (−) charged metabolites. Some metabolites have both positively and negatively charged groups and therefore appear in both fractions. (C) Select HILIC‐MS metabolite candidates which were significantly changed in the combination treated cells compared with DMSO. Relative metabolite abundances (to the DMSO control) are provided for reduced GSH and the amino acids; glutamate, aspartate and taurine. Error bars represent the standard error of the mean.

Overrepresentation (ORA) pathway analyses assess the overlap between differentially expressed genes and pathways, and is followed by statistical evaluation for significance.^66^ Differentially expressed genes in SK‐N‐BE(2)‐C and KELLY cells, and those in common between the two NB cell lines were assessed using ORA against the ReactomePA database^67^, ^68^, ^69^ (Tables S7 and S8). ORA analysis of the Reactome database revealed significant enrichments of pathways associated with ATF4‐pERK induced ER stress, alongside amino acid transport (Figure 5A). Taken together ORA analyses suggested an ER stress phenotype involving ATF4/mTOR signalling and amino acid transport was being induced by SGC0946 + GSK343 combination therapy.^70^

Next, microarray data from clinically annotated Kocak and SEQC NB patient cohorts were analysed using gene expression signatures created from the previous microarray analysis, where the 29 commonly differentially expressed genes upregulated by the combination therapy were used for the signature. Patient cohorts were first dichotomised by the median expression of this gene signature, followed by Kaplan–Meier analyses to assess their event‐free and overall survival over time. High expression of the combination drug response signature correlated with better event free and overall survival in NB patients of both cohorts (Figure S4C,D and Table S9). Furthermore, univariate CoxPH model of the gene signature, wherein patients were similarly dichotomised by median gene signature expression, revealed that patients with high signature expression were half as likely to experience an event or succumb to their disease, with hazard ratios ranging from 0.47 to 0.59. These data suggests that the transcriptional and phenotypic NB cell response caused by combination therapy predicted good patient outcomes.

### 3.5 Glutathione and specific amino acids are depleted by the combination of SGC0946 and GSK343

Our finding of several amino acid related pathways after combination treatment led us to perform metabolomic profiling on treated NB cells. Untargeted hydrophilic interaction chromatography mass spectrometry (HILIC‐MS) were conducted on SK‐N‐BE(2)‐C cell lysates after 6 h of combination treatment. HILIC‐MS can best identify highly polar metabolites which would be otherwise lost using other mass spectrometry methods.^71^ A total of 37 metabolites (33 unique metabolites) were detected between both positively and negatively charged HILIC‐MS fractions (Figure 5B and Table S10). Among those metabolites significantly changed by the combination therapy were glutathione (GSH) and several amino acids, such as glutamate, taurine and aspartate.

The combination reduced GSH by 2.1‐fold compared to the control treatment. GSH is known to protect NB cells from oxidative stress and when depleted can trigger cell death.^72^, ^73^ Hence the depletion of GSH facilitated by the combination of SGC0946 and GSK343 may, in part, explain their therapeutic synergy. Moreover, the ATF4 target gene, *CHAC1*, which was previously found to be upregulated by the combination therapy via microarray analysis, is a γ‐glutamyl cyclotransferase that degrades GSH, further supporting a mechanism of synergy involving GSH depletion.^74^

## 4 DISCUSSION

Several studies have now identified key epigenetic alterations that confer drug resistance in NB.^5^, ^75^ Targeting these epigenetic alterations has eventuated in marked anti‐tumour efficacy in models of NB.^3^, ^4^ Therefore, the identification of therapeutic synergy among these epigenetic‐targeted agents, as well as with other inhibitors targeting important oncogenic processes, may serve as a promising avenue to improving NB patient outcome. Here we show that combined inhibition of EZH2 and DOT1L HMTs with the small molecule inhibitors, SGC0946 and GSK343, synergistically drive a rapid ER stress response in NB cells, amino acid depletion, and reduced tumour growth in preclinical models suggesting a novel therapeutic strategy for NB.

The combination of GSK343 and SGC0946 was determined to be the most synergistic of the validated hits arising from the combination drug screen and it displayed preferential cytotoxicity against NB cells compared with normal fibroblasts. This is the first reported instance of synergy derived from targeting the two HMTs EZH2 and DOT1L. The HMT EZH2 is the catalytic subunit of the Polycomb‐repressive complex 2 (PRC2), which facilitates H3K27me3 modifications through its SET‐domain.^76^ DOT1L is the sole HMT responsible for H3K79me1–3 modifications, and unlike EZH2 does not have a SET‐domain.^77^ H3K27me3 modifications transcriptionally suppress many genes involved in cell proliferation, differentiation, and development.^78^ In contrast, H3K79me2 mediates the transcriptional activation of those genes.^79^, ^80^ Following Western blot analyses, decreased levels of H3K27me3 and H3K79me2 were observed 24 h after combined GSK343 and SGC0946 treatment, which is in line with previously published data for HMT inhibitors in NB.^10^, ^11^ From these findings, it was clear that dual HMT inhibitor therapy induced rapid transcriptional and metabolomic responses. Transcriptional profiling of treated cells revealed a rapid induction of ER stress, whereas metabolomic profiling revealed GSH and amino acid depletion after only 6 h of drug exposure. Given that H3K27me3 and H3K79me3 levels were not significantly altered at 6 h following treatment, the observed phenotypes could not be explained solely by perturbations to histone methylation. Indeed, a previous study had found that NB cell death induced by the EZH2 inhibitors, GSK126 and EPZ6438, could be abrogated by overexpressing a truncated version of EZH2 lacking the SET‐domain responsible for HMT activity.^55^ This suggested that EZH2 had critical functions, other than histone methylation, that promoted NB cell growth and that inhibitors of EZH2 may elicit their cytotoxicity independent of affecting histone methylation, as indicated in the present study. A key study in castration‐resistant prostate cancer revealed that EZH2 promoted tumorigenicity by acting as a cofactor to other transcription factors rather than canonically facilitating H3K27me3 modification, further supporting the notion that inhibiting EZH2 could elicit phenotypes independent of H3K27me3.^81^ Much like EZH2, DOT1L can bind with transcriptional cofactors, such as MYCN in NB cell lines, potentially leading to H3K79me2‐independent activity that promotes NB cell growth.^11^ A recent study assessing DOT1L function in embryonic stem cell differentiation found that DOT1L promoted transcriptional elongation independent of H3K79me1–3 modifications,^82^ further supporting the existence of H3K79 methylation‐independent functions of DOT1L. Aside from acting as transcriptional cofactors, HMTs can also methylate nonhistone proteins to mediate downstream signalling pathways.^83^ EZH2 is known to methylate transcription factors, (e.g. GATA4) and signalling mediators (e.g. RORα and STAT3).^84^, ^85^, ^86^, ^87^ In contrast, little is known about nonhistone substrates of DOT1L, though other closely related methyltransferase family members have been reported to methylate nonhistone proteins.^88^ Taken together, the combined inhibition of EZH2 and DOT1L HMTs may be defined by a mechanism that operates partially independent of histone methylation and instead relies on either, disrupting transcriptional coactivation/repression or reducing methylation of nonhistone proteins.

Following transcriptomic analyses of NB cell lines treated with the GSK343 and SGC0946 combination therapy, an ATF4‐mediated ER stress response was observed. Furthermore, ER stress signatures derived from these transcriptomic analyses predicted better prognosis in NB patient cohorts, suggesting that the observed ER stress induction was clinically favourable. ER stress and the subsequent unfolded protein response are regarded as synthetical lethal events in MYC‐driven cancers, such as NB.^89^, ^90^ Indeed, ATF4‐driven ER stress responses are known to be more efficiently induced in NB cells when either MYC or MYCN are expressed.^91^, ^92^ Treatment of NB cells with several targeted inhibitors had previously demonstrated ER stress induced cell death facilitated by ATF4 induction, following canonical PERK activation and eIF2α phosphorylation,^93^ thereby supporting ATF4‐PERK‐eIF2α driven ER stress as a canonical cell death signalling pathway in NB induced by the combination of SGC0946 and GSK343. However, a key limitation exists within the present study where protein levels of the ATF4‐PERK‐eIF2α axis were not assessed and could have provided further evidence for activation of the pathway. As a potential upstream regulator of ER stress, the mTORC1 signalling pathway was predicted to regulate ATF4 post‐translationally, which had been previously found in other cell contexts,^63^ though future studies would have to characterise this pathway after GSK343 and SGC0946 combination treatments. GSH and amino acid depletion were also observed, either, preceding or proceeding GSK343 and SGC0946 combination therapy‐induced ER stress. GSH depletion, using agents like L‐buthionine‐*S,R*‐sulfoximine (BSO), has been previously demonstrated to induce cell death in NB.^72^, ^94^ Although there is a lack of evidence in the literature linking GSH depletion and ER stress, the data presented in this study suggests that GSH depletion occurred as a consequence of ER stress. This is particularly evident in the observed upregulation of the ATF4‐regulated GSH degrading enzyme CHAC1 in response to SGC0946 and GSK343 treatment. In contrast to GSH depletion, amino acid depletion has been found to act as both a precursor to, and product of, ER stress responses in various other cell contexts.^61^, ^95^ Taken together, these findings suggest that mTORC1 activates ATF4‐PERK‐eIF2α driven ER stress, which is then followed by GSH and amino acid depletion prior to NB cell death.

Following in vivo administration of both SGC0946 and GSK343, a modest anti‐tumour efficacy was observed. SGC0946 and GSK343 have previously been administered as single agent therapies in xenografted Balb/c mouse models of glioma, cervical cancer or ovarian cancer via intraperitoneal injections of 5–10 mg/kg/day doses for 4–7 week periods.^52^, ^96^, ^97^ In the aforementioned single agent studies there were significant reductions in tumour growth after prolonged treatment, however, significant reductions were not observed for the single agents in the SK‐N‐BE(2)‐C xenograft model used in this study despite administering much higher doses of each compound (30 mg/kg/day) for a 3 week period. After the first 8 days of SGC0946 and GSK343 combination treatment, tumour growth in the SK‐N‐BE(2)‐C xenograft model was significantly reduced compared to the control or single agent groups, however by the 3 week time point the tumour size for the combination group was similar to that of the control and single agent groups. The observed difference to previous xenograft studies is most likely to have been attributed to the relatively shorter treatment window of 3 weeks due to the rapid tumour growth rate of SK‐N‐BE(2)‐C xenografts, compared to the xenograft models used in the previous single agent studies. The treatment window in the current combination study could be expanded by using a xenograft model with a much slower tumour growth rate. The poor bioavailability of SGC0946 and GSK343 may have also contributed to a lack of in vivo anti‐tumour activity and may explain discordance with the high cytotoxicity observed in vitro.^98^, ^99^ Taken together, the modest in vivo efficacy achieved by the combination of SGC0946 and GSK343 may reflect the NB xenograft animal model used in this study and the intrinsic pharmacokinetic properties of each compounds.

GSK343 and SGC0946 are considered to be tool compounds for investigating HMT‐related mechanisms and have limited clinical applications due to poor pharmacokinetic properties.^98^, ^99^ Given the modest anti‐tumour efficacy of GSK343 and SGC0946 in NB tumour models, alone or in combination, there is a need to consider more clinically advanced alternatives which are not limited by poor pharmacokinetic properties. Tazemetostat (EPZ‐6438) is a clinically advanced EZH2 inhibitor that has therapeutic efficacy against B‐cell non‐Hodgkin lymphoma and epithelioid sarcoma in several early Phase (I–II) clinical trials.^100^, ^101^, ^102^ Tazemetostat has also recently gained accelerated US FDA approval for use in adult and paediatric patients with advanced epithelioid sarcoma.^103^ However, preclinical testing of Tazemetostat as a single agent therapy in patient derived xenograft models of NB showed no significant extension of the overall survival of those mice,^104^ suggesting that combination therapies need to be designed for effective application of Tazemetostat in NB. In contrast GSK126 (GSK2816126), another inhibitor of EZH2, failed to elicit any marked anticancer response in patients with advanced haematological and solid tumours due to poor drug bioavailability,^105^ which bolsters the importance of drug properties in clinical translation. Pinometostat (EPZ‐5676) is a DOT1L inhibitor in phase I clinical trials for adult and paediatric MLL‐rearranged acute leukaemia,^106^, ^107^ however, it has not been tested in preclinical models of NB. Given that Tazemetostat and Pinometostat are clinically advanced alternatives to GSK343 and SGC0946, respectively, and have better pharmacokinetic properties, there is strong rationale for pursuing their combination in preclinical models, which may reveal higher anti‐tumour efficacy in NB animal models than what was observed in the present study.

## 5 CONCLUSION

In summary, we have identified and characterised a novel combination therapy that combines two HMT inhibitors targeting two functionally divergent histone marks. The combined inhibition of EZH2 and DOT1L HMTs using the small molecule inhibitors GSK343 and SGC0946, respectively, resulted in synergistic cell death in vitro as well as modest anti‐tumour efficacy in vivo. After transcriptomic and metabolic profiling of combination‐treated cells, ER stress phenotypes were observed that were either induced, or contributed to, GSH and amino acid depletion. Finally, ER stress signatures derived from SGC0946 and GSK343 combination therapy responses in vitro were found to associate with better NB patient prognoses. The finding of synergy between two independently acting HMT inhibitors is the first reported occurrence of such synergy and promotes further exploration of other HMT inhibitor combinations in both NB and other cancer settings. The modest anti‐tumour efficacy observed in NB animal models provides some promise for clinical translation. Finally, the phenotypic responses elicited by the combination therapy predict good patient outcome and can be used to inform NB prognoses or be utilised as a response biomarker to therapy in future studies.

## AUTHOR CONTRIBUTIONS

**Janith A. Seneviratne:** Conceptualization (equal); data curation (equal); formal analysis (equal); investigation (equal); methodology (equal); resources (equal); software (equal); supervision (equal); validation (equal); visualization (equal); writing – original draft (equal); writing – review and editing (equal). **Daenikka Ravindrarajah:** Conceptualization (equal); data curation (equal); formal analysis (equal); investigation (equal); methodology (equal); resources (equal); software (equal); supervision (equal); validation (equal); visualization (equal); writing – original draft (equal); writing – review and editing (equal). **Daniel R. Carter:** Conceptualization (equal); data curation (equal); formal analysis (equal); funding acquisition (equal); methodology (equal); project administration (equal); resources (equal); software (equal); supervision (equal); validation (equal); visualization (equal); writing – review and editing (equal). **Vicki Zhai:** Data curation (supporting); investigation (supporting); validation (supporting). **Amit Lalwani:** Data curation (supporting); investigation (supporting). **Sukriti Krishan:** Resources (supporting). **Anushree Balachandran:** Data curation (supporting); investigation (supporting). **Ernest Ng:** Data curation (supporting); investigation (supporting). **Ruby Pandher:** Resources (supporting); software (supporting). **Matthew Wong:** Resources (supporting). **Tracy L. Nero:** Resources (supporting); writing – review and editing (supporting). **Shudong Wang:** Resources (supporting); writing – review and editing (supporting). **Murray D. Norris:** Funding acquisition (supporting); project administration (supporting); resources (supporting). **Michelle Haber:** Funding acquisition (supporting); project administration (supporting); resources (supporting). **Tao Liu:** Project administration (supporting); resources (supporting). **Michael W. Parker:** Resources (supporting); writing – review and editing (supporting). **Belamy B. Cheung:** Conceptualization (equal); funding acquisition (equal); project administration (equal); resources (equal); supervision (equal); writing – review and editing (equal). **Glenn M. Marshall:** Conceptualization (equal); funding acquisition (equal); project administration (equal); resources (equal); supervision (equal); writing – review and editing (equal).

## FUNDING INFORMATION

This work was supported by Program Grants (Glenn M. Marshall, Murray D. Norris, Michelle Haber) from the National Health and Medical Research Council (NHMRC) Australia (APP1016699), Cancer Institute NSW (10/TPG/1‐13), Cancer Council NSW (PG‐11‐06) and an Australia Research Training Program Scholarship, UNSW Sydney, Australia (Janith A. Seneviratne). This work was also supported by a NHMRC Project Grant (APP1125171; Glenn M. Marshall, Belamy B. Cheung), Cancer Council NSW Project Grants (RG21‐08; Belamy B. Cheung and RG214491; Belamy B. Cheung), TKCP and Neuroblastoma Australia Research Grant (RG231618 and RG231619; Belamy B. Cheung). This work was also supported by Cancer Institute NSW Program Grant (RG211809; Glenn M. Marshall), TKCP Project Grant (RG213283; Glenn M. Marshal) and NHMRC Synergy Grant (RG220538; Glenn M. Marshall). This work was also supported by grant awarded through the Priority‐driven Collaborative Cancer Research Scheme and co‐funded by Cancer Australia and The Kids' Cancer Project (1,123,235; D.R. Carter, G.M Marshall).

## CONFLICT OF INTEREST STATEMENT

The authors do not have a conflict of interest to declare.

## ETHICS STATEMENT

The animal studies were approved by the UNSW Animal Ethics Committee (ACEC Approval Number: 19/88B).

## Supporting information


Data S1.

## Data Availability

The data that supports the findings of this study are available in the supplementary material of this article and are available from the corresponding author upon reasonable request.
