# Supplementary material for: Combined inhibition of histone methyltransferases EZH2 and DOT1L is an effective therapy for neuroblastoma
Source: Cancer Med. 2024 Nov 5;13(21):e70082. doi: 10.1002/cam4.70082 (PMC11538032; doi:10.1002/cam4.70082)
Supplement: Supplementary file 1 — Data S1. [file CAM4-13-e70082-s001.pdf]

Table S1

Compound	Target	IC_{50} (μM)	Drug Doses (μM)					
			0	1	2	3	4	5
JQ1	BRD4	0.23	0	0.075	0.15	0.3	0.6	1.2
13-*cis*-RA	RA (agonist)	ND	0	1.25	2.5	5	10	20
SAHA	pan-HDAC (Class I & II)	2.06	0	0.375	0.75	1.5	3	6
GSK343	EZH2	12.05	0	1.25	2.5	5	10	20
SGC0946	DOT1L	20.26	0	1.25	2.5	5	10	20
GSK-LSD1	LSD1	ND	0	1.25	2.5	5	10	20
5-AZA-dC	DNMT1	ND	0	1.25	2.5	5	10	20
MLN8237	AURKA	0.087	0	0.0125	0.025	0.05	0.1	0.2
NVP-BEZ235	PI3K/mTOR	ND	0	0.005	0.01	0.02	0.04	0.08
Crizotinib	ALK/ROS1	0.53	0	0.15	0.3	0.6	1.2	2.4
Mafosfamide	DNA Alkylating	ND	0	1.25	2.5	5	10	20
CBL0137	FACT	0.15	0	0.05	0.1	0.2	0.4	0.8
CDKI-71	CDK9	0.096	0	0.025	0.05	0.1	0.2	0.4

Table S2

Target gene	Forward primer (5'-3')	Reverse primer (5'-3')
CHAC1	CTG GGC CTC TTA CCC ACT TG	GGG ACA GAC TGG GAA GGT TG
DDIT4	GTG CCC TCC AAG ACA GAG AC	CAC CCG CAC ACA ACT CAA TG
OPRL1	GTA GTA GAT GGG GGT GTG GC	GGC TGC CGT AGA TAA CCT CC
SESN2	CCG CTA CAT GAC CTG ACT CC	CTG CAC ATC ACA CAC AAG CC
SLC7A5	CCT CCA GCA TGT AGG CGT AG	CCG AGG AGA AGG AAG AGG C
SLC7A11	CTG CTT TGG CTC CAT GAA CG	GGC AGA TTG CCA AGA TCT CAA G

Figure S1

(A)

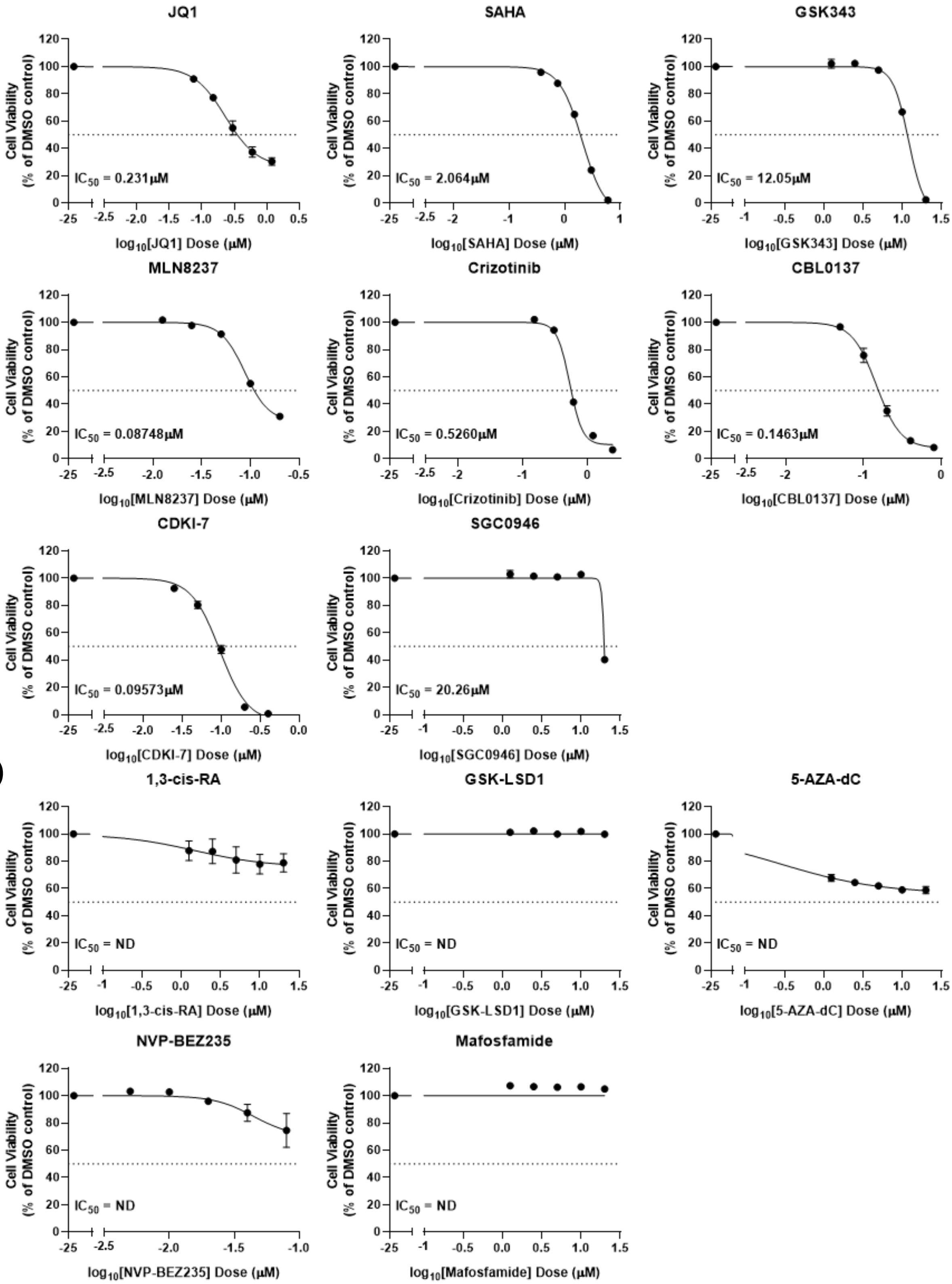
JQ1
IC50 = 0.231μM
log10[JQ1] Dose (μM)
SAHA
IC50 = 2.064μM
log10[SAHA] Dose (μM)
GSK343
IC50 = 12.05μM
log10[GSK343] Dose (μM)
MLN8237
IC50 = 0.08748μM
log10[MLN8237] Dose (μM)
Crizotinib
IC50 = 0.5260μM
log10[Crizotinib] Dose (μM)
CBL0137
IC50 = 0.1463μM
log10[CBL0137] Dose (μM)
CDKI-7
IC50 = 0.09573μM
log10[CDKI-7] Dose (μM)
SGC0946
IC50 = 20.26μM
log10[SGC0946] Dose (μM)
(B)
1,3-cis-RA
IC50 = ND
log10[1,3-cis-RA] Dose (μM)
GSK-LSD1
IC50 = ND
log10[GSK-LSD1] Dose (μM)
5-AZA-dC
IC50 = ND
log10[5-AZA-dC] Dose (μM)
NVP-BEZ235
IC50 = ND
log10[NVP-BEZ235] Dose (μM)
Mafosfamide
IC50 = ND
log10[Mafosfamide] Dose (μM)
Cell Viability (% of DMSO control)

Table S3

Drug Combination	HSA		BLISS	
	**Max**	**Total**	**Max**	**Total**
JQ1+13-*cis*-RA	0.18	1.49	0.09	-0.75
JQ1+SAHA	0.41	2.76	0.23	1.21
JQ1+GSK343	0.27	0.95	0.08	0.2
JQ1+SGC0946	0.1	0.21	0.16	0.98
JQ1+GSK-LSD1	0.06	-0.1	0.08	-0.01
JQ1+5-aza-dc	0.24	3.11	0.07	-0.05
JQ1+MLN8237	0.33	3.84	0.18	2.35
JQ1+NVP-BEZ235	0.12	0.56	0.1	0.09
JQ1+Crizotinib	0.42	2.85	0.23	1.65
JQ1+Mafosfamide	0.05	-0.08	0.1	0.78
JQ1+CBL0137	0.19	1.8	0.09	0.17
JQ1+CDKI-7	0.53	4.88	0.4	3.26
13-*cis*-RA+SAHA	0.19	2.16	0.21	1
13-*cis*-RA+GSK343	0.32	2.88	0.3	2.03
13-*cis*-RA+SGC0946	0.17	1.74	0.25	2.03
13-*cis*-RA+GSK-LSD1	0.12	0.4	0.16	0.96
13-*cis*-RA+5-aza-dc	0.24	3.51	0.09	0.28
13-*cis*-RA+MLN8237	0.17	0.74	0.14	-0.16
13-*cis*-RA+NVP-BEZ235	0.13	0.72	0.2	2.18
13-*cis*-RA+Crizotinib	0.08	-0.55	0.05	-1.69
13-*cis*-RA+Mafosfamide	0.05	-0.84	0.1	0.51
13-*cis*-RA+CBL0137	0.24	0.63	0.19	0.28
13-*cis*-RA+CDKI-7	0.16	1.19	0.1	0.01
SAHA+GSK343	0.31	1.53	0.21	1.16
SAHA+SGC0946	0.35	1.46	0.26	1.55
SAHA+GSK-LSD1	0.14	1.26	0.14	0.88
SAHA+5-AZA-DC	0.24	1.88	0.09	-0.46
SAHA+MLN8237	0.45	2.43	0.21	1.47
SAHA+NVP-BEZ235	0.15	-0.46	0.11	-0.85
SAHA+Crizotinib	0.52	2.89	0.34	2.03
SAHA+Mafosfamide	0.08	-0.59	0.12	0.19
SAHA +CBL0137	0.24	1.49	0.11	0.33
SAHA +CDKI-7	0.36	2.18	0.23	1.18
GSK343+SGC0946	0.78	4.52	0.77	5.97
GSK343+GSK-LSD1	0.11	0.69	0.11	0.64
GSK343+5-AZA-DC	0.4	2.98	0.24	1.76
GSK343+MLN8237	0.28	1.04	0.18	1.53
GSK343+NVP-BEZ235	0.18	0.61	0.14	0.61

Table S3 (continue)

SGC0946+Crizotinib	0.18	0.92	0.2	0.6
SGC0946+Mafosfamide	0.05	-0.63	0.1	-0.75
SGC0946+CBL0137	0.34	0.95	0.28	0.92
SGC0946+CDKI-7	0.22	0.42	0.12	0.3
GSK-LSD1+5-AZA-DC	0.08	0.23	0.05	-0.03
GSK-LSD1+MLN8237	0.07	-0.32	0.16	1.32
GSK-LSD1+NVP-BEZ235	0.1	-0.74	0.11	-0.42
GSK-LSD1+Crizotinib	0.1	-0.38	0.1	-0.51
GSK-LSD1+Mafosfamide	0.11	0.48	0.27	2.85
GSK-LSD1+CBL0137	0.07	-0.14	0.1	-0.08
GSK-LSD1+CDKI-7	0.02	-1.06	0.01	-1.04
5-AZA-DC+MLN8237	0.14	1.4	0.11	0.22
5-AZA-DC+NVP-BEZ235	0.19	1.81	0.08	-0.01
5-AZA-DC+Crizotinib	0.13	0.59	0.07	-0.63
5-AZA-DC+Mafosfamide	0.03	-0.11	0.07	0.71
5-AZA-DC+CBL0137	0.14	1.05	0.04	-1.15
5-AZA-DC+CDKI-7	0.14	1.15	0.03	-0.9
MLN8237+NVP-BEZ235	0.22	1.66	0.28	2.84
MLN8237+Crizotinib	0.23	0.56	0.13	-0.01
MLN8237+Mafosfamide	0.1	-0.32	0.16	0.31
MLN8237+CBL0137	0.27	1.42	0.09	0.37
MLN8237+CDKI-7	0.2	0.78	0.02	-0.25
NVP-BEZ235+Crizotinib	0.13	0.14	0.11	-0.33
NVP-BEZ235+Mafosfamide	0.11	-0.24	0.2	1.5
NVP-BEZ235+CBL0137	0.12	0.62	0.06	0.19
NVP-BEZ235+CDKI-7	0.12	0.03	0.04	-0.85
Crizotinib+Mafosfamide	0.04	0.13	0.18	1.61
Crizotinib+CBL0137	0.3	1.43	0.14	0.63
Crizotinib+CDKI-7	0.12	-0.55	0.03	-1.24
Mafosfamide+CBL0137	0.1	0.25	0.19	1.62
Mafosfamide+CDKI-7	0.05	-0.1	0.13	0.73
CBL0137+CDKI-7	0.13	0.48	0.01	-0.42

Table S4

NB cell lines
Normal cells

Cell Line	BLISS Average	CI IC$_{50}$	CI IC$_{75}$	CI IC$_{90}$	CI Dm	CI m	CI r
BE(2)-C	0.11	1.03	0.84	0.69	5.73	4.49	0.97
KELLY	0.014	0.94	0.71	0.56	6.8	5.50	0.94
SK-N-FI	0.0076	0.63	0.37	0.22	9.66	5.46	0.87
SH-EP	0.14	1.12	1.04	0.97	5.33	4.55	0.97
SH-SY5Y	0.094	1.03	0.85	0.70	5.57	4.22	0.95
SK-N-AS	0.045	0.68	0.36	0.20	7.27	4.21	0.96
CHP-134	0.082	0.90	0.67	0.50	6.26	5.23	0.95
LAN-1	0.079	0.93	0.66	0.47	6.01	4.37	0.92
MRC-5	-0.13	1.13	1.06	1.22	9.41	3.24	0.83
WI-38	-0.15	0.94	0.65	0.46	7.64	3.69	0.92

Figure S2

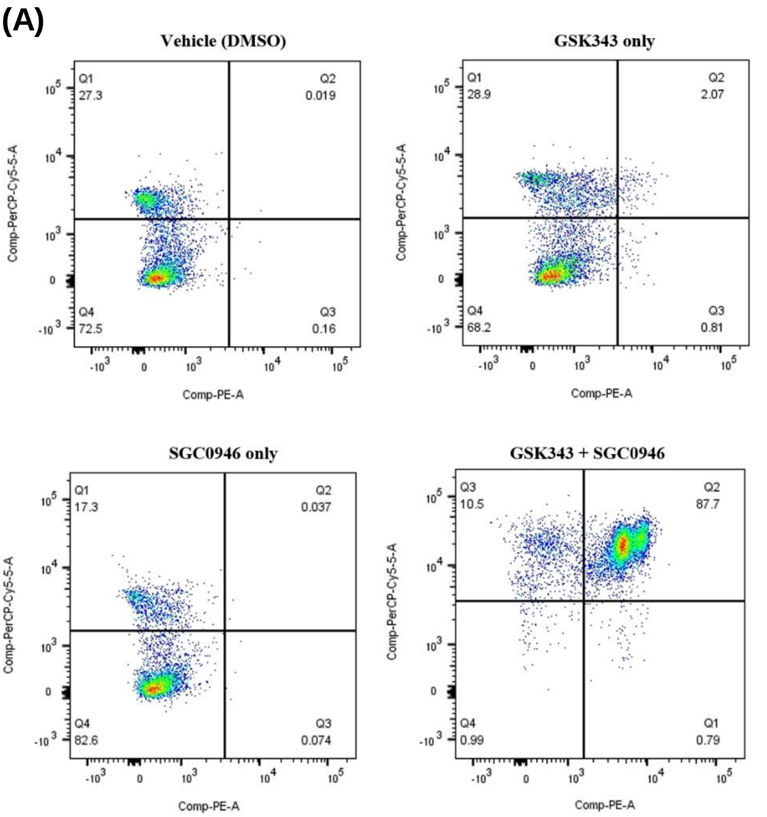
(A)
Vehicle (DMSO)
Q1 27.3
Q2 0.019
Q3 0.16
Q4 72.5
GSK343 only
Q1 28.9
Q2 2.07
Q3 0.81
Q4 68.2
SGC0946 only
Q1 17.3
Q2 0.037
Q3 0.074
Q4 82.6
GSK343 + SGC0946
Q3 10.5
Q2 87.7
Q4 0.99
Q1 0.79
Comp-PerCP-Cy5-5-A
Comp-PE-A

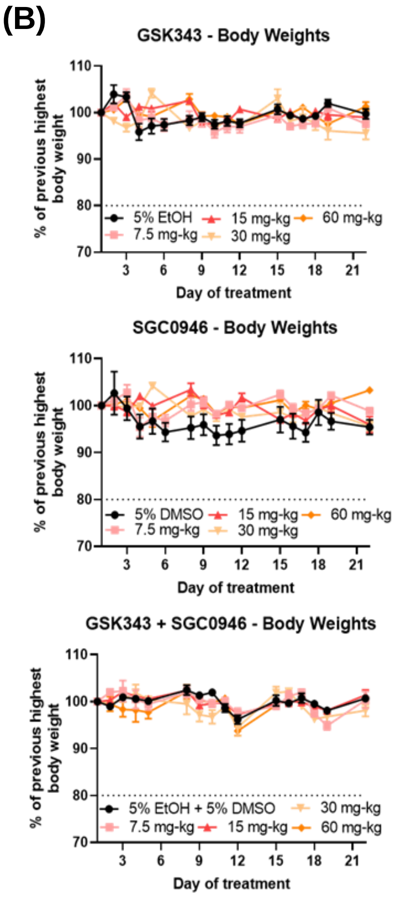
(B)
GSK343 - Body Weights
SGC0946 - Body Weights
GSK343 + SGC0946 - Body Weights
% of previous highest body weight
Day of treatment
5% EtOH
5% DMSO
5% EtOH + 5% DMSO
7.5 mg-kg
15 mg-kg
30 mg-kg
60 mg-kg

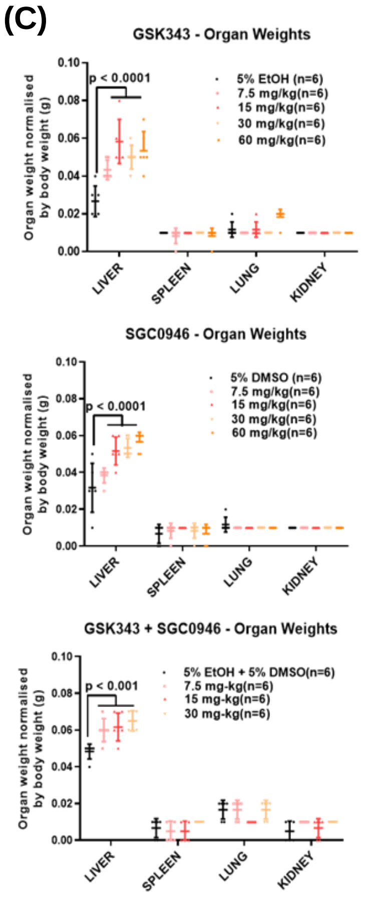
(C)
GSK343 - Organ Weights
SGC0946 - Organ Weights
GSK343 + SGC0946 - Organ Weights
Organ weight normalised by body weight (g)
p < 0.0001
p < 0.001
5% EtOH (n=6)
5% DMSO (n=6)
5% EtOH + 5% DMSO(n=6)
7.5 mg/kg(n=6)
15 mg/kg(n=6)
30 mg/kg(n=6)
60 mg/kg(n=6)
7.5 mg-kg(n=6)
15 mg-kg(n=6)
30 mg-kg(n=6)
LIVER
SPLEEN
LUNG
KIDNEY

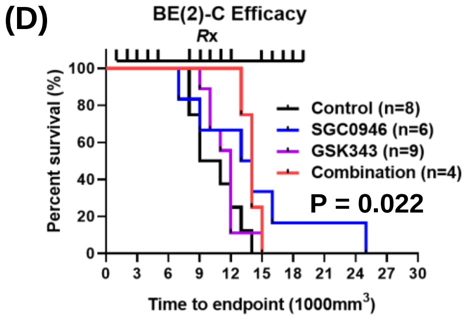
(D)
BE(2)-C Efficacy
Rx
Percent survival (%)
Control (n=8)
SGC0946 (n=6)
GSK343 (n=9)
Combination (n=4)
P = 0.022
Time to endpoint (1000mm³)

Figure S3

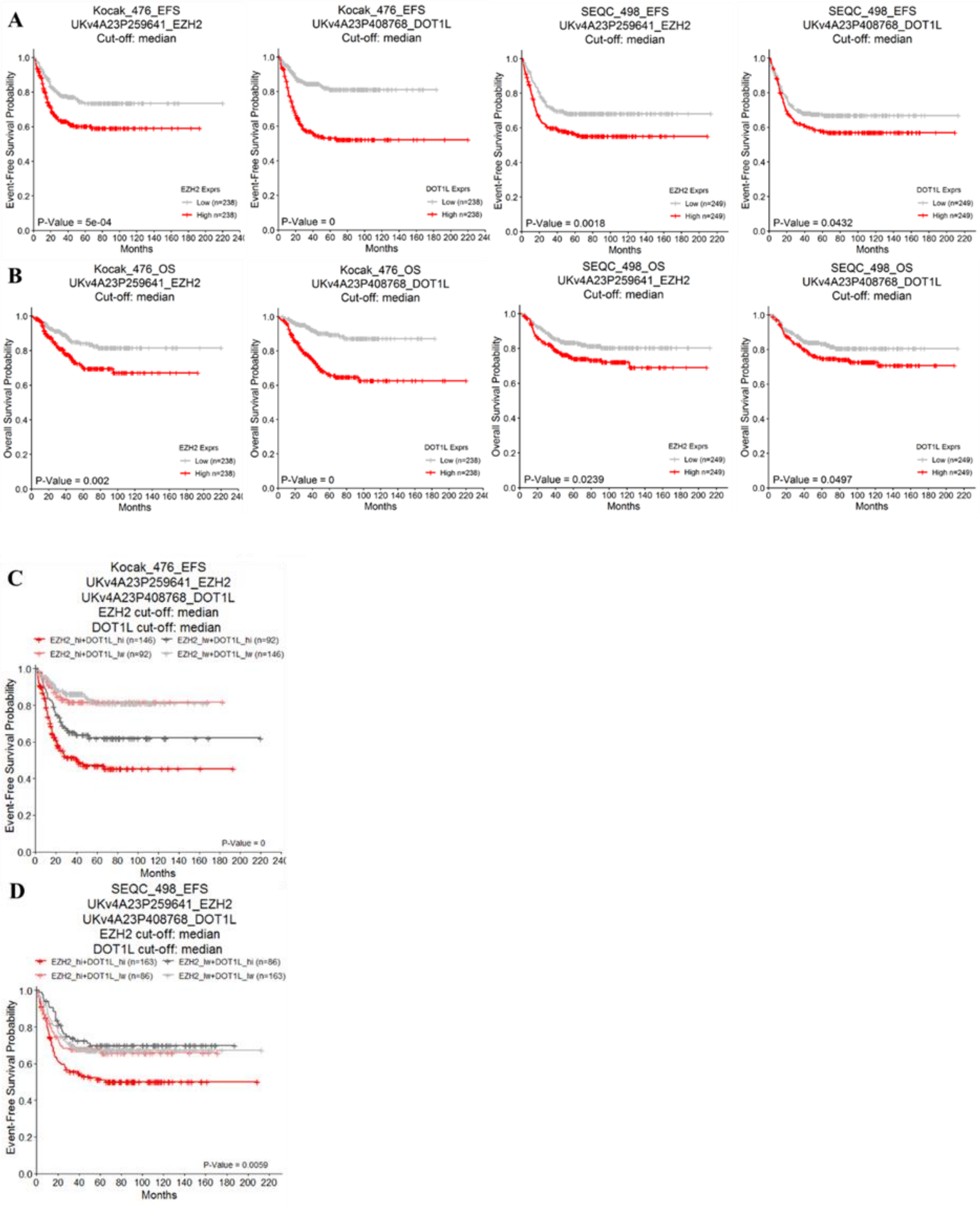
A
Kocak_476_EFS
UKv4A23P259641_EZH2
Cut-off: median
Event-Free Survival Probability
EZH2 Exprs
Low (n=238)
High n=238)
P-Value = 5e-04
Months
Kocak_476_EFS
UKv4A23P408768_DOT1L
Cut-off: median
DOT1L Exprs
Low (n=238)
High n=238)
P-Value = 0
SEQC_498_EFS
UKv4A23P259641_EZH2
Cut-off: median
Low (n=249)
High n=249)
P-Value = 0.0018
SEQC_498_EFS
UKv4A23P408768_DOT1L
Cut-off: median
P-Value = 0.0432
B
Kocak_476_OS
UKv4A23P259641_EZH2
Cut-off: median
Overall Survival Probability
P-Value = 0.002
Kocak_476_OS
UKv4A23P408768_DOT1L
Cut-off: median
P-Value = 0
SEQC_498_OS
UKv4A23P259641_EZH2
Cut-off: median
P-Value = 0.0239
SEQC_498_OS
UKv4A23P408768_DOT1L
Cut-off: median
P-Value = 0.0497
C
Kocak_476_EFS
UKv4A23P259641_EZH2
UKv4A23P408768_DOT1L
EZH2 cut-off: median
DOT1L cut-off: median
EZH2_hi+DOT1L_hi (n=146)
EZH2_lw+DOT1L_hi (n=92)
EZH2_hi+DOT1L_lw (n=92)
EZH2_lw+DOT1L_lw (n=146)
P-Value = 0
D
SEQC_498_EFS
UKv4A23P259641_EZH2
UKv4A23P408768_DOT1L
EZH2 cut-off: median
DOT1L cut-off: median
EZH2_hi+DOT1L_hi (n=163)
EZH2_lw+DOT1L_hi (n=86)
EZH2_hi+DOT1L_lw (n=86)
EZH2_lw+DOT1L_lw (n=163)
P-Value = 0.0059

Figure S4

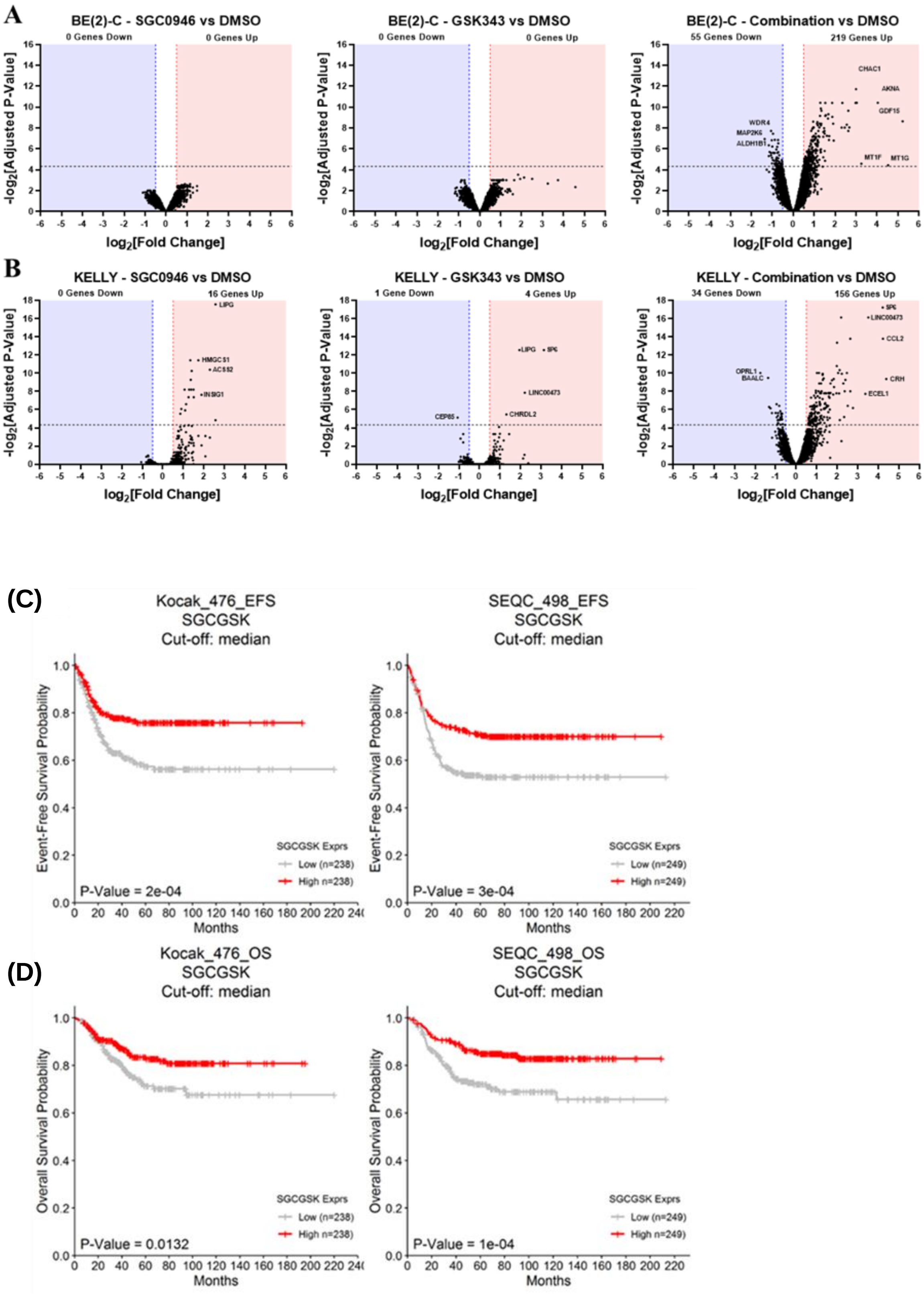
A
BE(2)-C - SGC0946 vs DMSO
0 Genes Down
0 Genes Up
BE(2)-C - GSK343 vs DMSO
0 Genes Down
0 Genes Up
BE(2)-C - Combination vs DMSO
55 Genes Down
219 Genes Up
CHAC1
AKNA
GDF15
MT1F
MT1G
WDR4
MAP2K6
ALDH1B1
-log2[Adjusted P-Value]
log2[Fold Change]
B
KELLY - SGC0946 vs DMSO
0 Genes Down
16 Genes Up
LIPG
HMGCS1
ACSS2
INSIG1
KELLY - GSK343 vs DMSO
1 Gene Down
4 Genes Up
LIPG
SP6
LINC00473
CHRDL2
CEP85
KELLY - Combination vs DMSO
34 Genes Down
156 Genes Up
SP6
LINC00473
CCL2
CRH
ECEL1
OPRL1
BAALC
(C)
Kocak_476_EFS
SGCGSK
Cut-off: median
Event-Free Survival Probability
SGCGSK Exprs
Low (n=238)
High n=238)
P-Value = 2e-04
Months
SEQC_498_EFS
SGCGSK
Cut-off: median
Event-Free Survival Probability
SGCGSK Exprs
Low (n=249)
High n=249)
P-Value = 3e-04
Months
(D)
Kocak_476_OS
SGCGSK
Cut-off: median
Overall Survival Probability
SGCGSK Exprs
Low (n=238)
High n=238)
P-Value = 0.0132
Months
SEQC_498_OS
SGCGSK
Cut-off: median
Overall Survival Probability
SGCGSK Exprs
Low (n=249)
High n=249)
P-Value = 1e-04
Months

Table S5

	Kocak (n=476)				SEQC (n=498)			
	EFS		OS		EFS		OS	
	HR	P-Val	HR	P-Val	HR	P-Val	HR	P-Val
DOT1L (UV)	3.2(2.2-4.7)	4.70E-11	3.6(2.2-5.8)	1.70E-08	1.4(1-1.8)	0.043	1.5(1-2.2)	0.049
EZH2 (UV)	1.8(1.3-2.5)	0.00047	1.9(1.3-2.9)	0.002	1.6(1.2-2.1)	0.0019	1.6(1.1-2.3)	0.024
DOT1L (MV)	2.1(1.4-3.1)	0.00032	1.8(1.1-3)	0.022	1.2(0.9-1.7)	0.18(n.s.)	1.2(0.8-1.7)	0.44 (n.s.)
EZH2 (MV)	1.5(1.1-2.2)	0.014	1.6(1-2.5)	0.032	1.6(1.2-2.1)	0.0022	1.5(1-2.3)	0.031

Table S6

Gene Symbol	SK-N-BE(2)-C Combination vs DMSO			KELLY Combination vs DMSO		
	log_2FC	P-Value	Adj. P-Value	log_2FC	P-Value	Adj. P-Value
CHAC1	3.01	6.0E-09	2.9E-04	1.59	1.2E-05	6.4E-03
SLC7A11	2.63	5.0E-07	1.2E-03	1.65	8.5E-05	2.1E-02
DDIT4	2.56	3.2E-06	3.1E-03	2.37	7.7E-06	5.3E-03
SESN2	2.35	1.4E-07	7.4E-04	1.19	2.4E-04	3.7E-02
CCNG2	1.72	1.2E-05	6.6E-03	1.63	2.1E-05	9.6E-03
PDE4B	1.54	1.6E-07	7.4E-04	0.98	2.5E-05	1.1E-02
CEBPB	1.34	2.8E-06	2.9E-03	0.84	3.4E-04	4.6E-02
RHEBL1	1.20	2.8E-05	1.0E-02	0.95	2.8E-04	4.1E-02
VEGFA	1.16	2.6E-05	9.5E-03	0.94	2.0E-04	3.5E-02
SLC3A2	1.16	2.2E-05	8.6E-03	1.14	2.6E-05	1.1E-02
ADAMTS9	1.13	3.0E-04	3.4E-02	1.13	3.1E-04	4.3E-02
SLC7A5	1.08	1.4E-05	7.0E-03	1.34	1.2E-06	1.9E-03
HERPUD1	1.02	2.2E-05	8.6E-03	0.79	2.9E-04	4.1E-02
LONRF1	1.01	1.9E-04	2.8E-02	0.98	2.4E-04	3.7E-02
VLDLR	1.00	2.4E-04	3.1E-02	1.36	1.1E-05	6.2E-03
RIMKLB	0.99	1.3E-05	6.9E-03	0.74	2.3E-04	3.7E-02
HBP1	0.95	8.6E-05	1.9E-02	1.03	4.1E-05	1.4E-02
COL4A3BP	0.93	1.6E-04	2.6E-02	0.89	2.4E-04	3.7E-02
INSM1	0.92	1.7E-04	2.6E-02	1.15	1.8E-05	8.6E-03
SHMT2	0.91	5.8E-06	4.1E-03	0.78	3.3E-05	1.3E-02
GAB2	0.90	1.1E-04	2.1E-02	0.90	1.1E-04	2.4E-02
NR4A2	0.88	2.1E-05	8.6E-03	0.68	2.7E-04	4.0E-02
YPEL2	0.81	1.1E-04	2.1E-02	0.71	3.6E-04	4.8E-02
BNIP3L	0.79	2.0E-04	2.9E-02	0.92	4.4E-05	1.4E-02
ING4	0.72	1.3E-04	2.3E-02	0.75	8.9E-05	2.2E-02
DEPTOR	0.66	3.9E-04	4.0E-02	0.76	1.2E-04	2.6E-02
CLCN6	0.66	1.9E-04	2.8E-02	0.88	1.1E-05	6.2E-03
ANKMY2	0.58	3.8E-04	3.9E-02	0.59	3.0E-04	4.2E-02
TPST1	0.57	4.4E-04	4.2E-02	0.68	7.9E-05	2.1E-02
WDR4	-1.06	6.8E-06	4.7E-03	-0.77	1.7E-04	3.2E-02
OPRL1	-1.12	8.4E-05	1.9E-02	-1.75	6.8E-07	1.4E-03

Table S7

Pathway	Database	Gene Ratio	P-Val	Adj. P-Val
PERK regulates gene expression	Reactome	0.08	0.0025	0.042
ATF4 activates genes in response to endoplasmic reticulum stress	Reactome	0.08	0.0018	0.04
Amino acid transport across the plasma membrane	Reactome	0.12	5.9E-05	0.003
Transport of inorganic cations/anions and amino acids/oligopeptides	Reactome	0.12	0.002	0.04
mTOR signaling pathway	KEGG	0.26	2.3E-05	0.0013
Neutral amino acid transmembrane transporter activity	GO	0.1	1.9E-05	0.0033
Amino acid transmembrane transporter activity	GO	0.1	2.4E-04	0.017
Antiporter activity	GO	0.1	4E-04	0.012

Table S8

Pathway	Database	BE(2)-C			KELLY		
		NES	P-Val	Adj.P-Val	NES	P-Val	Adj.P-Val
KRIGE_AMINO_ ACID_DEPRIVATION	C2	2.9	0.002	0.03	2.7	0.0015	0.018
ATF3_Q6	C3	1.73	0.0015	0.025	2.33	0.0014	0.021
CREB_Q2	C3	1.69	0.0014	0.025	2.27	0.0011	0.021
CREB_Q4	C3	1.68	0.0015	0.025	2.25	0.0011	0.021
ATF1_Q6	C3	1.65	0.0015	0.024	2.20	0.0012	0.021
CREBP1CJUN_01	C3	1.60	0.0015	0.024	2.13	0.0011	0.021
TGACGTCA_ATF3_Q6	C3	1.45	0.0059	0.037	2.23	0.0012	0.021
CREB_01	C3	1.41	0.0087	0.044	2.20	0.0011	0.021
CREB_Q2_01	C3	1.49	0.0043	0.036	2.10	0.0012	0.021
ATF4_Q2	C3	1.60	0.0015	0.025	1.98	0.0011	0.021
CREBP1_Q2	C3	1.43	0.0074	0.042	2.15	0.0011	0.021
ATF_01	C3	1.50	0.0058	0.037	1.99	0.0011	0.021
MTOR_UP.N4.V1_UP	C6	2.22	0.0015	0.013	2.21	0.0012	0.010
MTOR_UP.N4.V1_DN	C6	2.07	0.0015	0.013	1.68	0.0037	0.022
HALLMARK_MTORC1_ SIGNALING	H	1.76	0.0015	0.011	2.27	0.0012	0.006

Table S9

	Kocak (n=476)				SEQC (n=498)			
	EFS		OS		EFS		OS	
	HR	P-Val	HR	P-Val	HR	P-Val	HR	P-Val
SGCGSK (UV)	0.53(0.38-0.74)	0.00026	0.59(0.39-0.9)	0.013	0.58(0.43-0.78)	0.00027	0.47(0.31-0.7)	0.00014

Table S10

Metabolite	HILIC Fraction	log_2FC (vs Control)			Comb. Vs Con.	
		GSK343	SGC0946	Comb.	P-Val	Adj. P-Val
Cystathionine*	-	-0.39	0.088	-0.72	0.0028	0.049
Cystathionine*	+	-0.37	-0.022	-0.72	0.0028	0.049
1-Methylguanine	+	-0.40	-0.52	-0.70	0.0048	0.049
Pantothenic acid	-	-0.61	-0.20	-0.62	0.0054	0.049
Uridine 5'-diphospho glucuronic acid	-	-0.44	-0.23	0.26	0.0078	0.059
Phosphocreatine	-	-1.04	0.81	-0.94	0.011	0.059
L-Glutathione (reduced)	+	-0.52	0.18	-1.05	0.012	0.059
Cyclic ADP-ribose	-	-0.28	0.26	-0.052	0.014	0.059
L-Aspartic acid	+	-0.58	0.27	-0.81	0.014	0.059
3,5-Dimethoxyaniline	+	0.063	0.17	2.46	0.019	0.072
Adenosine	+	0.66	-1.47	0.54	0.024	0.078
L-Glutamic acid	+	-0.29	-0.24	-0.40	0.025	0.078
Taurine	-	-0.58	0.011	-0.78	0.029	0.083
